# A third somatomotor representation in the human cerebellum

**DOI:** 10.1152/jn.00165.2022

**Published:** 2022-09-21

**Authors:** Noam Saadon-Grosman, Peter A. Angeli, Lauren M. DiNicola, Randy L. Buckner

**Affiliations:** ^1^Department of Psychology, Center for Brain Science, Harvard University, Cambridge, Massachusetts; ^2^Athinoula A. Martinos Center for Biomedical Imaging, Massachusetts General Hospital, Charlestown, Massachusetts; ^3^Department of Psychiatry, Massachusetts General Hospital, Charlestown, Massachusetts

**Keywords:** fMRI, motor, somatosensory

## Abstract

Seminal neurophysiological studies in the 1940s discovered two somatomotor maps in the cerebellum—an inverted anterior lobe map and an upright posterior lobe map. Both maps have been confirmed in the human using noninvasive neuroimaging with additional hints of a third map within and near to the cerebellar vermis. Here, we sought direct evidence for the third somatomotor map by using intensive, repeated functional MRI (fMRI) scanning of individuals performing movements across multiple body parts (tongue, hands, glutes, and feet). An initial discovery sample (*n* = 4, 4 sessions per individual including 576 separate blocks of body movements) yielded evidence for the two established cerebellar somatomotor maps, as well as evidence for a third discontinuous foot representation within the vermis. When the left versus right foot movements were directly contrasted, the third representation could be clearly distinguished from the second representation in multiple individuals. Functional connectivity from seed regions in the third somatomotor representation confirmed anatomically specific connectivity with the cerebral cortex, paralleling the patterns observed for the two well-established maps. All results were prospectively replicated in an independent dataset with new individuals (*n* = 4). These collective findings provide direct support for a third somatomotor representation in the vermis of the cerebellum that may be part of a third map. We discuss the relations of this candidate third map to the broader topography of the cerebellum as well as its implications for understanding the specific organization of the human cerebellar vermis where distinct zones appear functionally specialized for somatomotor and visual domains.

**NEW & NOTEWORTHY** A third somatomotor representation exists in the vermis of the human cerebellum. Evidence for this elusive representation arises specifically from mapping the foot. Separate foot representations distinguish the third from the nearby second somatomotor representation. A third somatomotor representation in the posterior vermis supports a large-scale organization hypothesis in which the cerebellum possesses three sets of roughly homotopic representations of the full cerebrum.

## Graphical abstract

**Figure:**
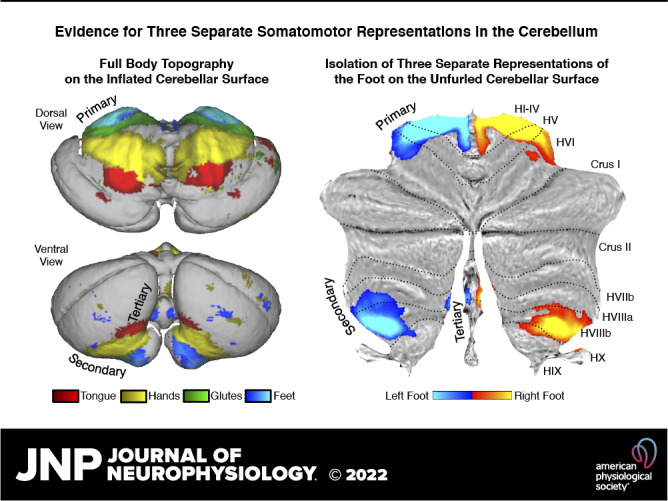

## INTRODUCTION

A somatomotor topography was first described in the anterior lobe of the cerebellum by Adrian (1). In cats and monkeys, Adrian recorded afferent discharges reaching the cerebellum following electrical stimulation to the cerebral cortex, tactile stimulation, movements of joints, and stretching of muscles. He discovered that body parts are topographically organized in an inverted fashion beginning with the hindlimb represented in lobules III and HIII, the forelimb in lobules IV, V, and HIV and HV, and the face in lobules VI and HVI at the posterior border of the anterior lobe. A year after Adrian’s seminal observations, Snider and Stowell (2) confirmed the anterior lobe somatomotor topography and discovered a second discontinuous body map in the posterior lobe that was upright rather than inverted. They recorded cerebellar-evoked potentials, also in cats and monkeys, following tactile stimulation. In the paramedian lobule (lobules HVII, HVIII), the hindlimb representation was found posteriorly, the face anteriorly and the forelimb in between (2; see also Ref. 3).

A half century after their discovery, the anatomical basis of the two separate somatomotor maps was revealed using transneuronal viral tracing techniques. A barrier to measuring anatomical connectivity between the cerebral cortex and the cerebellum was that the pathways are polysynaptic and did not yield to traditional monosynaptic anatomical tracing techniques (4–6). Using polysynaptic tracing techniques based on specific strains of herpes and rabies viruses, Strick and coworkers (7–9) demonstrated that the hand region of the monkey’s cerebral motor cortex projects to and receives input from cerebellar lobules V-VI in the anterior lobe and HVIIb-HVIII in the posterior lobe, consistent with the two well-established somatomotor maps.

The two distinct somatomotor maps have been repeatedly observed in humans using noninvasive neuroimaging techniques evoked by movements (10–15) and through tactile stimulation (16–18). Functional connectivity between cerebral motor regions and the cerebellum in task-free data also reveals the two maps (13, 15, 19, 20). Thus, multiple approaches have converged to suggest that the cerebellum possess two distinct somatomotor body maps.

Beyond these two established somatomotor body maps, we have also postulated the existence of a third, smaller body map in the posterior extent of the cerebellum within and near to the vermis (13). There are multiple motivations for this hypothesis. In comprehensive mapping efforts that have explored both somatomotor and nonsomatomotor regions of the cerebellum, an orderly progression of zones is found along the anterior-to-posterior axis of the cerebellum variably described as a sequence of networks or a gradient (13, 19–21). The progression begins with the inverted somatmotor map in the anterior lobe of the cerebellum and then progresses through multiple sensory-motor and higher cognitive networks ending at the Crus I/II border. The progression of networks inverts at the Crus I/II border and proceeds in mirror-reversed order through the posterior lobe of the cerebellum ending on the well-established upright somatomotor map. Although these two gradients alone capture many features of the observed organization of the cerebellum, they fail to explain a key feature.

Specifically, cerebral networks associated with higher-order functions display representations in the posterior lobe of the cerebellum near lobule IX, which would be expected if there was a third gradient, including somatomotor representation. In particular, the network referred to in the human literature as the default network possesses a robust representation in lobule IX (13, 20, 22, 23). Tasks targeting social inferences, which increase response in the default network, also reveal a response in lobule IX (14, 15, 24). Furthermore, in their seminal work, Kelly and Strick (9) noted that Crus I/II receive prefrontal projections but also described a smaller group of neurons in lobules IX/X. The labeled neurons fall within the region of the suggested tertiary association map in the posterior end of the cerebellum (see Fig. 1 in Ref. 15). One possibility is that a third map represents cognitive networks but not motor networks, or what Guell et al. (15) refer to as the “double motor/triple nonmotor representation hypothesis.” The alternative possibility is that the cerebellum possesses a third somatomotor map that is small and challenging to identify.

Hints of a third somatomotor representation exist in the literature. In an early electrophysiological study, Dow and Anderson (25) documented responses to tactile stimulation in the pyramis (part of the posterior vermis) of rats. With transneuronal viral tracing techniques, Coffman et al. (26) showed that the cerebellar vermis is a target of projections from motor regions in the cerebral cortex of monkeys. More broadly, this finding challenged longstanding concepts about functional organization of the cerebellum. The vermis was considered part of the “spino-cerebellum” as it is the target of ascending spinal pathways while the lateral cerebellar cortex is part of the “cerebro-cerebellum” due to dense interconnections with the cerebral cortex. Coffman et al. (26) discovered that the density of projections from motor regions in the cerebral cortex to the vermis is as great as the density of projections to the lateral cerebellar cortex.

In humans, one of the first studies to use fMRI to map the cerebellum noted that there might be a third somatomotor representation in the vermis (11). However, this suggestion was made with caution due to the small sample size and mixed results. In our prior well-powered, group-averaged study, a small medial region within and near to the vermis of the cerebellum was linked to cerebral somatomotor networks (see the transverse section at *Z* = −38 and the coronal section at *Y* = −68 in Fig. 8 of Ref. 13). But the representation in the vermis could not be disambiguated from the adjacent zones of the cerebellum attributed to the established second somatomotor map. Similarly, Guell et al. (15) noted a candidate motor representation of the foot near the vermis but with sufficient location uncertainty to downplay the result.

A definitive demonstration of a third somatomotor map, if one exists, faces multiple challenges. First, the hypothesized map is expected to be found in a small region within the already small cerebellum. Second, the inferior portions of the cerebellum near the vermis have low signal-to-noise ratio (SNR) in fMRI compared with the cerebral cortex.^1^ Finally, a third somatomotor map is expected to be found in close spatial proximity to the second map, making it difficult to dissociate between the two posterior lobe representations.

In the present work, we took a number of steps to overcome these challenges. First, we acquired a substantial amount of data within individual participants during task-based motor movements to boost SNR. Each participant was scanned on four days with six motor runs per day, for a total of 576 separate blocks of motor movements (96 blocks for each of six separate motor conditions). Second, to avoid between-individual spatial blurring, each participant was initially analyzed separately (20, 28). Third, we used an experimental paradigm that targeted movement across the body parts, including the hands and feet on both sides. Given the orientation of the second somatomotor map, one but not both of the hand and foot representations should juxtapose one another, allowing the other body part to demonstrate separation of the second and hypothesized third maps. As the results will reveal, we were able to identify a clear third representation in most of the individuals studied, with movements of the feet being the critical condition to differentiate the second and third somatomotor representations.

## METHODS

### Participants

Eight healthy adults, aged 19–25 [means = 22.4 yr (SD = 2.6), 2 men, 7 right-handed], were recruited from the Boston area. Four participants contributed data to the initial Discovery sample, and four participants contributed data to the Replication sample. All participants were screened to exclude a history of neurological and psychiatric illness or ongoing use of psychoactive medications. Paid participants provided written informed consent through a protocol approved by the Institutional Review Board of Harvard University.

### MRI Data Acquisition

Scanning was conducted at the Harvard Center for Brain Science using a 3 T Siemens Magnetom Prisma-fit MRI scanner and a 64-channel phased-array head-neck coil (Siemens Healthcare, Erlangen, Germany). Inflatable padding provided comfort and helped immobilize the head. Participants viewed a rear-projected display through a mirror attached to the head coil. Before each session, the screen presentation was adjusted so that the center point could be comfortably viewed without any eye strain. Participants’ eyes were monitored and video-recorded using the Eyelink 1000 Core Plus with Long-Range Mount (SR Research, Ottawa, Ontario, Canada), and alertness was scored during each functional run.

All participants were scanned across four MRI sessions on separate nonconsecutive days. Six task-based runs were acquired each day where participants made active movements (motor runs) as well as two runs where participants fixated on a centrally presented black crosshair on a light gray background (fixation runs). In total, each participant had 24 motor and 8 fixation runs. Four participants (*S5*–*S8*) were scanned in an additional session not used here.

Functional data used a multiband gradient-echo echo-planar pulse sequence sensitive to blood oxygenation level-dependent (BOLD) contrast (29–32), generously provided by the Center for Magnetic Resonance Research (CMRR) at the University of Minnesota. The four Discovery sample participants (*S1*–*S4*) were scanned with two different spatial resolutions: 1.8 mm (2 sessions) and 2.4 mm (2 sessions) isotropic voxels (order of sessions was balanced across participants). All sessions of the four Replication sample participants (*S5*–*S8*) were scanned with 2.4 mm isotropic voxels. Acquisition parameters for 1.8 mm resolution: TR = 2,000 ms, TE = 30 ms, flip-angle = 80°, matrix 122 × 122 × 87 (FOV = 220 × 220), multislice three times acceleration. Two hundred eleven volumes were acquired for each fixation run and 214 volumes for each motor run. Acquisition parameters for 2.4 mm resolution: TR = 1,000 ms, TE = 33 ms, flip-angle = 64°, matrix 92 × 92 × 65 (FOV = 221 × 221), multislice five times acceleration. Four hundred twenty-two volumes were acquired for each fixation run and 428 volumes for each motor run. The two resolutions in the Discovery sample were acquired to explore the possibility of higher (1.8 mm) resolution disambiguating functional details better than lower (2.4 mm) resolution. For the present purposes, the two resolutions were combined and smoothed with a uniform kernel. The first two sessions of *S5* and the first session of *S6* were acquired in a different FOV (211 × 211); therefore, the matrix for both BOLD runs and field maps was: 88 × 88 × 65 and BOLD TE = 32.6 ms. The change in FOV, which occurred to accommodate larger heads, did not affect the quality of registration or impact the analyses in any way we could detect.

A T1-weighted structural image was obtained in each session using a rapid multiecho magnetization-prepared rapid gradient echo (MPRAGE) three-dimensional sequence (33): TR = 2,200 ms, TE = 1.57, 3.39, 5.21, 7.03 ms, TI = 1,100 ms, flip-angle = 7°, voxel size 1.2 mm, matrix 192 × 192 × 176, in-plane generalized auto-calibrating partial parallel acquisition (GRAPPA) 4 times acceleration. In each session, two dual gradient-echo B0 field maps were also acquired to correct for susceptibility-induced gradient inhomogeneities with slice spatial resolution matched to the BOLD sequence (1.8 mm or 2.4 mm). Other field map parameters included: 1.8 mm resolution—TE = 4.73 ms, 7.19 ms, TR = 806 ms, flip angle = 55°; 2.4 mm resolution—TE = 4.45 ms, 6.91 ms, TR = 295 ms, flip angle = 55°.

Exclusion criteria included a maximum absolute motion of no more than 1.8 mm. One motor run was excluded for *S3*, two motor runs for *S4*, one motor and two fixation runs for *S6*, and one fixation run for *S8* due to motion. One border case motor run for *S4* (with maximum absolute motion of 1.88 mm) was included as the motion estimate was mostly the result of a linear drift. In three motor runs of *S3*, an artifact in the form of a peak translation (in the *z* axis and to a lesser extent in the *y*-axis) was spotted in approximately the same time point across these three runs. Maximum absolute motion did not exceed the set threshold for these runs (0.72, 1.01, 0.85 mm), but we conservatively excluded them from analysis. Runs were excluded based on BOLD data quality before examination of task response patterns to avoid bias.

### Motor Task

Participants performed a blocked-task paradigm consisting of six types of movements that targeted four separate parts along the anterior-posterior body axis: *1*) tongue: participants moved their tongue from right to left, touching their upper premolar teeth; *2* and *3*) right and left hands: participants moved their fingers alternating between tapping the thumb with the index finger and the thumb with the middle finger; *4*) glutes: participants contracted and relaxed their gluteal muscles; and *5* and *6*) right and left feet: participants alternated dorsiflexion and plantarflexion of their toes.

Each movement was performed repeatedly in 10-s active movement blocks. Prior to a movement block, a visual cue of a drawn body part with a text label was presented for 2 s, informing the participant to initiate one of the six movement types. Then, the fixation crosshair was presented with a black circle surround that repeatedly flashed on and off to pace the movements (1 s on then 1 s off). The onset of the black circle cued movement of the tongue to the right, thumb to index finger, glutes contraction, and toes plantarflexion. The black circle turning off cued movement of the tongue to the left, thumb to middle finger, glutes relaxation, and toes dorsiflexion. The word “End” was presented for 1 s at the end of each movement block to instruct the participant to stop, and there was a 1-s fixation gap before the onset of the next movement cue.

Twenty-four movement blocks (4/body part) occurred within each motor run, with 16-s blocks of passive fixation providing a break after each set of six movement blocks. All runs began and ended with fixation yielding five fixation blocks per run. Six separate runs with distinct orders of movement conditions were performed on each day. Counterbalancing of the movement conditions across the six runs on each day ensured that each condition appeared exactly once in each of the run positions.

Participants extensively practiced the intended movements before the first scanning session. First, participants practiced remotely during a consent and training session, after watching a video demonstrating how to execute the movements. Emphasis was placed on how to localize each movement in a subtle way to avoid head motion. Next, when participants arrived for their first session, they practiced again while lying on the scanner bed outside of the bore and then a final time inside the bore. To further reduce extraneous motion, participants’ legs were supported in a semiflexed position using an ergonomic knee-to-ankle cushion.

### Data Processing

A custom analysis pipeline for individualized data processing was used, as described in detail in the study by Braga et al. (27). Briefly, the pipeline combines tools from FreeSurfer (34), FSL (35), and AFNI (36) to align data within an individual across runs and sessions to a high-resolution output target (1 mm isotropic) using a single interpolation to minimize spatial blurring. Five different registration matrices were combined for the single interpolation: *1*) a motion correction matrix for each volume to the run’s middle volume [linear registration, 6 degrees of freedom (DOF); MCFLIRT, FSL], *2*) a matrix for field-map-unwarping the run’s middle volume (FUGUE, FSL), *3*) a matrix for registering the field-map-unwarped middle volume to a mean BOLD template (12 DOF; FLIRT, FSL), *4*) a matrix for registering the mean BOLD template to the participant’s native space T1 image (6 DOF; using boundary-based registration, FSL), and *5*) a matrix for registering the native space T1 to the MNI152 1 mm atlas (nonlinear registration; FNIRT, FSL). The mean BOLD template was created by taking the mean of all field-map-unwarped middle volumes after registration to an upsampled (1.2 mm), unwarped mid-volume template (temporary target, selected from a low motion run acquired close to a field map). The native space template was one of the participant’s T1 structural images, upsampled to 1 mm isotropic resolution. Given that multiple structural images were available for each individual, a single reference image was chosen that possessed a robust estimate of the pial and white-matter boundaries (as constructed by FreeSurfer recon-all).

Data were checked for registration errors. In cases of suboptimal registration, a different mid-volume temporary template was chosen, or a different/additional field map was used for unwarping (since two field maps were acquired in each session). Before the calculation of these matrices, for each BOLD run, the first 12 volumes were discarded for T1 equilibration (6 volumes in 1.8 mm resolution). For the participants (*S1*–*S4*) that had two spatial resolutions (two sessions with 1.8 mm isotropic voxels and two sessions with 2.4 mm isotropic voxels), each resolution was processed separately and then combined. All functional data were smoothed in the volume with a 3-mm full width at half maximum (FWHM) kernel.

Data were analyzed in the standard space of the MNI152 atlas within each participant individually. Thus, all idiosyncratic details within the individual were preserved. The use of the atlas space allowed the separate participants to be examined in a spatially registered framework and also allowed their data to be projected to a flat representation of the cerebellar cortex to aide visualization of patterns.

### Visualization on the Cerebellar Surface

To visualize the spatial extent of maps in the cerebellum, the data were projected onto flat and inflated representations of the cerebellar surface [similar to our prior work in Xue et al. (20)]. These representations were created by Diedrichsen and Zotow (14) and utilized within the spatially unbiased infratentorial template (SUIT) toolbox (http://www.diedrichsenlab.org/imaging/suit_fMRI.htm). The projection method uses an approximate surface of the gray and white matter defined on the SUIT template (here specifically, MNI152, normalized by FSL). The vertices on the two surfaces come in pairs. When projecting, the algorithm samples the data along the line connecting the two vertices and then takes the mean of these numbers to determine the value of the vertex.

The surface representation was used for display purposes only. The surface is not unique to each individual. In addition, the flat surface may not be optimal for the cerebellar vermis. To enable flattening, a cut was made between the posterior vermis and lobules HVII-HIX that can introduce distortions to the vermal region. Nonetheless, the surface representation can display the individual participant’s topographic details in a uniform, comprehensive display framework. To avoid ambiguities in anatomical localization, key findings were also displayed across multiple slices through the cerebellar volume, including the vermis.

### Motor Task Analysis

Motor run data in MNI152 atlas space were initially analyzed within each individual separately. A high-pass filter with a 100-s (0.01 Hz) cut-off was applied to remove low-frequency drifts within each run. Run-specific general linear model (GLM, using FEAT; FSL) analyses were performed, including the six movement conditions as regressors of interest. The onsets of the six movement blocks were modeled 1 s before the initiation of movement (1 s after the appearance of the visual cue). The duration of each event block was set to 10 s. Events were modeled with a canonical hemodynamic response function (double-γ) along with its temporal derivative. GLM analyses resulted in voxel-level β values for each movement.

### Somatomotor Topography

Initial analyses focused on estimating the somatomotor map across the four body parts in the cerebral cortex and separately in the cerebellum. In total there were six conditions because there were two (right and left) movements for the hands and feet. A contrast map was computed for each of the movements by subtracting all of the other five movements’ β values (weighted so that the target movement β map was balanced with the subtracted movements, here achieved by multiplying the target movement β map by 5 before subtracting the 5 other βeta values). This was done in each motor run separately and then averaged across runs (including averaging across resolutions in *S1*–*S4*). To visualize somatomotor topography, a winner-take-all map was generated. Each voxel was assigned with the highest β value across all movement contrasts (one versus all the others) and was given the color of the corresponding movement: tongue—red, right and left hand—yellow, glutes—green, and right and left foot—blue. In this visualization, right and left representations were not distinguished. Maps were visualized separately for each individual in the volume and also projected onto the SUIT flatmap template of the cerebellum. Maps were thresholded within individuals to best capture somatomotor topography, separately for the cerebrum and for the cerebellum.

### Focus on the Hand and Foot Representations

A critical aspect of the design is the ability to contrast left and right movements separately for the hands and feet. This enables direct contrast of the left and right movements within each body part, allowing a clean estimate of the representation of the body part because nonspecific noise and other features of the data are largely nulled [paralleling the strategy we have repeatedly used in group-averaged functional connectivity data (13, 23)]. Contrast maps were averaged across runs (and across resolutions in *S1*–*S4*). Contrast maps were projected onto a flatmap template of the cerebellar surface and thresholded within individuals.

### Seed-Based Functional Connectivity Analysis

To assess representations’ specificity, we conducted a seed-based functional connectivity analysis. This analysis should not be considered independent as we utilized all available data, including the motor task runs used to make the contrast maps. Specifically, in addition to the 24 motor runs (across 4 sessions) of each individual, we used the eight fixation runs (2 per session), resulting in 32 possible runs. After quality control exclusions, *S3* had 28 runs (193.3 min), *S4* had 30 runs (207.2 min), *S6* had 29 runs (200.5 min), and *S8* had 31 runs (214.2 min). All other participants utilized all 32 runs (221.1 min). Note that unlike typical task-free data (fixation), the present data included substantial modulation due to active task demands.

Preprocessing included all steps as used for the prior task-based analyses but also regression of nuisance variables (3dTproject, AFNI) as is typical in functional connectivity analysis (whole brain, ventricular, and deep cerebral white matter mean signals, six motion parameters, and their temporal derivatives). The residual BOLD data were then band-pass filtered at 0.01–0.1 Hz and smoothed with a 3 mm FWHM kernel paralleling the task-based analysis.

Seed regions were defined in the right and left primary, secondary, and tertiary somatomotor cerebellar representations. For each seed region, an MNI152 coordinate was selected in the volume to best capture the center of the representation in the right versus left hand and foot contrast maps. In one individual, *S8*, we selected right and left foot tertiary coordinates to be similar to the ones of other individuals given the lack of detectable representations.

Seed regions were defined with a 2-mm radius centered at the selected MNI152 coordinate (33 voxels). For each run, the Pearson’s correlation was computed between the averaged time course of the seed region and that of all other voxels. The functional connectivity values were averaged across all runs (and resolutions for *S1*–*S4*) after being Fisher’s r-to-z transformed.

### Discovery and Replication

A key aspect of our methods was prospective replication. Data from the initial four participants were fully analyzed and graphed (Discovery sample, *S1*–*S4*) before any analysis was attempted on the second, independent group of participants (Replication sample, *S5*–*S8*).

## RESULTS

### Somatomotor Topography is Detected within the Primary Motor Cortex

**Figure 1.**
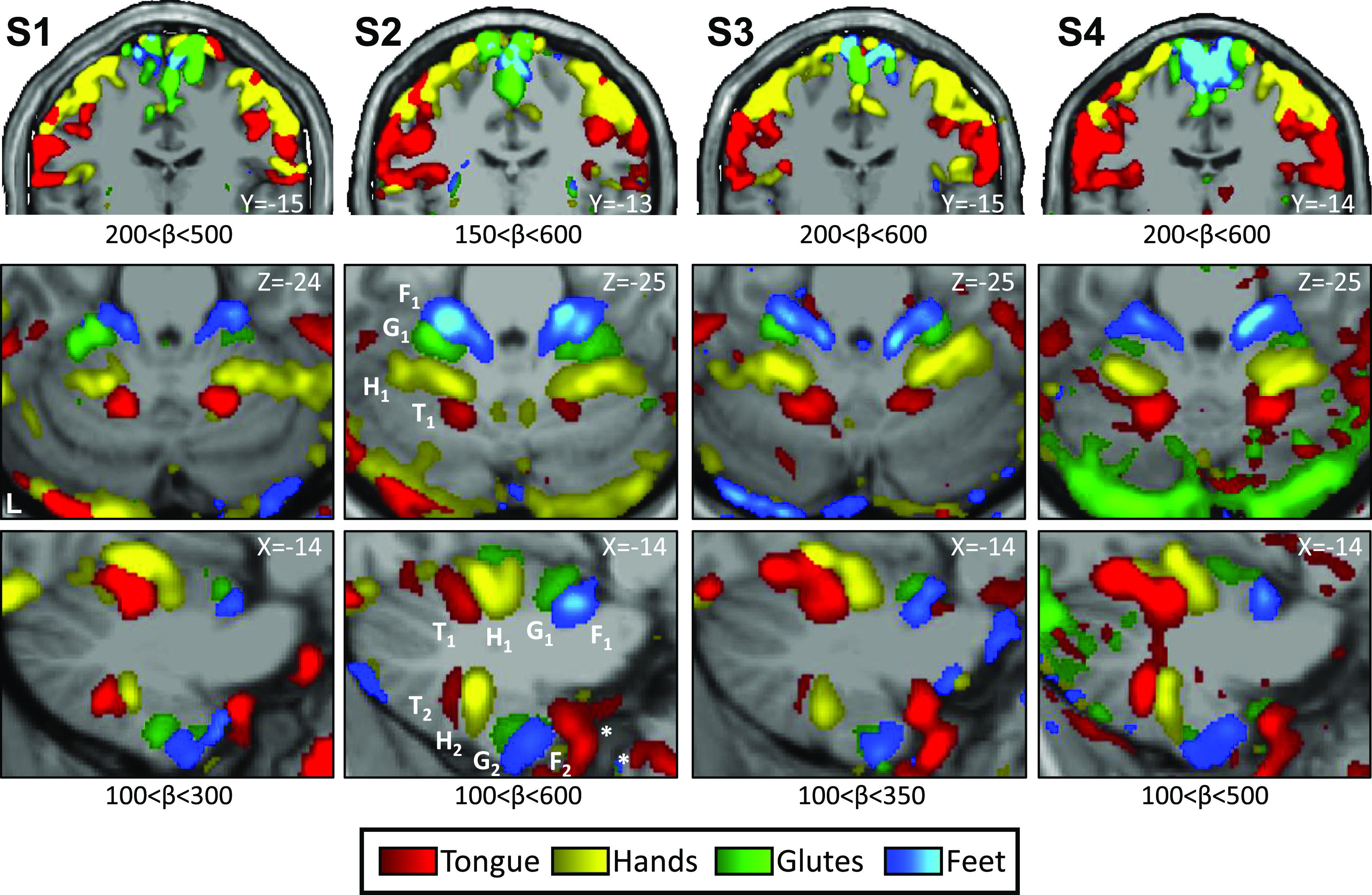
Somatomotor topography in the cerebral cortex and cerebellum is evident in individual participants. Winner-take-all maps of active movements are displayed for four body parts along the anterior-posterior body axis: tongue (red), right and left hands (yellow), glutes (green), and right and left feet (blue). Each column displays a separate participant (*S1*–*S4*). β values are thresholded to best capture topography, separately for the cerebral cortex and cerebellum. In each participant, a clear body topography is evident by the order tongue-hand-glutes-foot in the primary motor cortex, M1 (lateral to medial, *top row*), cerebellar anterior lobe (*middle* and *bottom rows*), and in the cerebellar posterior lobe (*bottom row*). The primary cerebellar representation (T1-H1-G1-F1) is inverted to the secondary representation as labeled in *S2* (T2-H2-G2-F2). Note the tongue assignment anterior to the secondary representation (in all participants, marked with an asterisk in *S2*) is outside the cerebellum and likely results from a motion artifact. Also note that the cerebral coronal sections also include parts of the supplementary motor area (SMA) along the midline, which are continuous to M1 (foot and glutes) and that the chosen slice for *S3* shows the order most clearly in the right hemisphere. Coordinates indicate the section level in the space of the MNI152 atlas. The color bars represent β values. L indicates left.

The body map in the primary motor cortex (M1, precentral gyrus) is well established. The anterior (head) to posterior (foot) axis is topographically organized as one moves from the lateral extent of the motor strip to the midline (37). Data from each individual revealed the full M1 body topography: tongue-hand-glutes-foot, lateral to medial as expected (Fig. 1, *top*). Although not common in the literature, contraction and relaxation of the gluteal muscles was included to evoke a representation of the trunk. As predicted by the body topography, representation of the glutes fell between the hand and the foot. The observed M1 motor topography validates the experimental paradigm and the processing methods applied.

### Somatomotor Topography is Detected in Both of the Established Maps within the Cerebellum

The two well-established cerebellar somatomotor maps were apparent in all participants (Fig. 1, *middle* and *bottom*). The ordering of body parts was inverted between the two maps. In the anterior lobe, the foot was anterior to the tongue and in the posterior lobe the tongue was anterior to the foot (Fig. 1, *bottom*, highlighted in *S2*). In both maps, the glutes representation falls between the hand and foot, filling in a gap often observed in motor body maps (e.g., see Fig. 3 in Ref. 20), although more apparent in the primary map than the secondary and cleaner for some participants (*S1* and *S2*) than others (*S3* and *S4*). The current data thus reveal strong and reliable evidence for the anterior lobe and posterior lobe somatomotor maps.

One other aspect of the results (Fig. 1, *bottom*) is that the tongue movements induced noise centered outside of the cerebellum (but extending into the cerebellum). Given the sensitivity of fMRI to motion artifact, it is not surprising that movements within the field of view produce artifact. Fortunately, the predicted tongue representation is spatially distant from the region of artifact and thus easy to distinguish.

**Figure 2.**
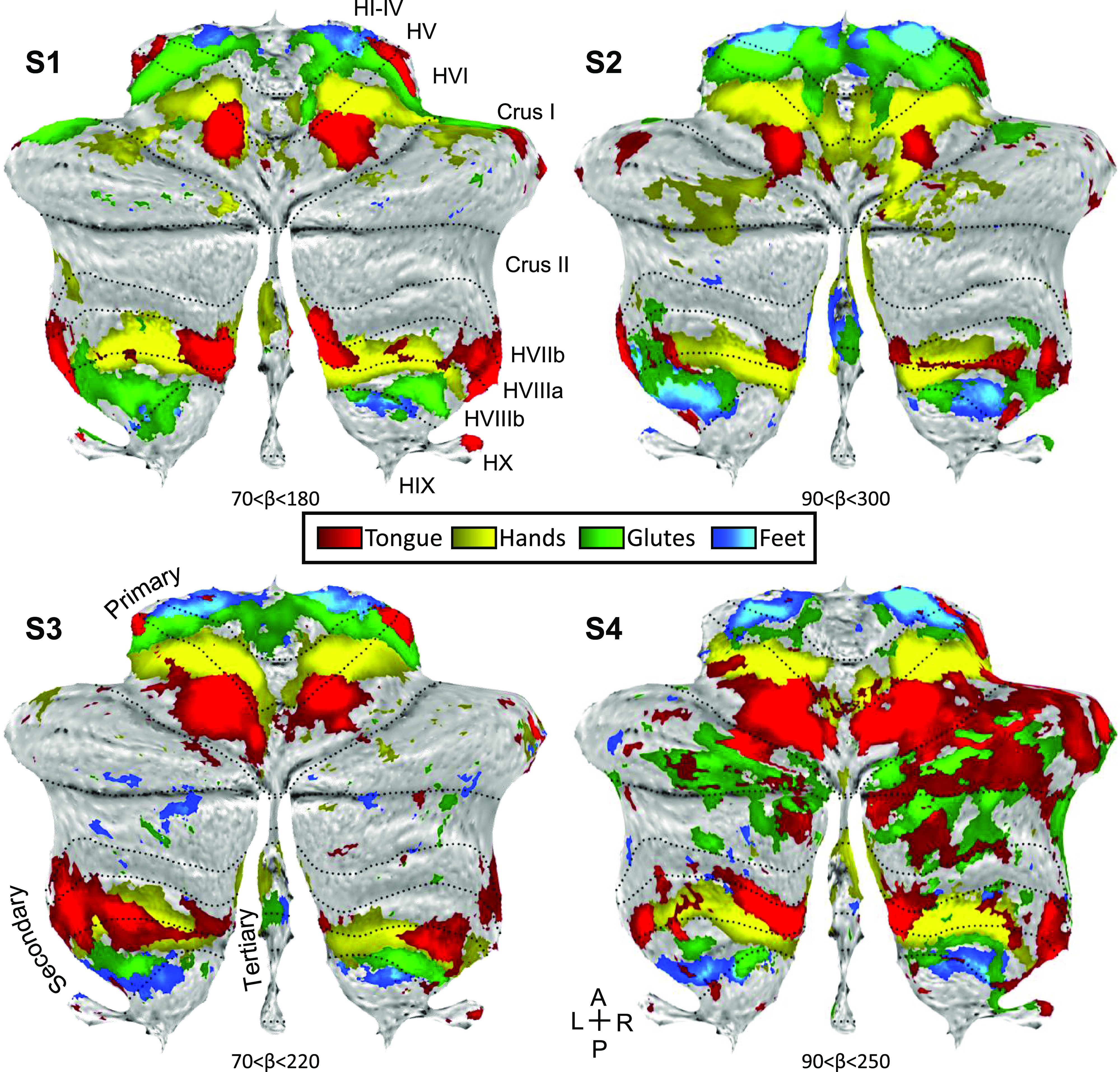
Somatomotor topography projected onto a flatmap of the cerebellum reveals details. Winner-take-all maps of active movements are displayed for four body parts along the anterior-posterior body axis projected onto flatmaps using the SUIT toolbox: tongue (red), right and left hands (yellow), glutes (green), and right and left feet (blue). Each panel displays a separate participant (*S1*–*S4*). In each participant, the primary and secondary somatomotor maps are apparent in the anterior and posterior lobes of each hemisphere corresponding to the anterior-posterior body axis. Also note the medial representations within the vermis (glutes and feet in *S2* and *S3*, and hands in all four individuals). These may be hints for the hypothesized tertiary body representation in the cerebellum. Glutes and tongue responses in the Crus I/II of S4 are likely due to motion artifacts that are more common during these movements. Dotted lines indicate approximate lobule boundaries with lobules labeled for *S1*. A, anterior; L, left; P, posterior; R, right.

To visualize the entirety of the somatomotor cerebellar topography, maps with representations of the four body part movements were projected onto a flatmap view of the cerebellum (Fig. 2). In this view, the inversion of the two map representations is clear. Note that the second map evolves also over the medial-lateral axis, with anterior-medial (close to the vermis) tongue representation distant from the posterior-lateral foot representation.

Figure 2 also shows provisional evidence for a third map within and near to the vermis. The hand representation in all four participants extends into the region of the vermis, as does the foot and glutes representations in *S2* and *S3*. This feature is consistent with the hypothesized third (tertiary) somatomotor map of the cerebellum. However, idiosyncrasies in the data, variability across participants, and the adjacency to the secondary representation make it uncertain. To further explore the possibility of a third map, we focused specifically on hand and foot movements where direct contrasts between the right and left movements were possible.

### The Hand Representation in the Cerebellum is Consistent with, but Does Not Differentiate, A Third Somatomotor Map

**Figure 3.**
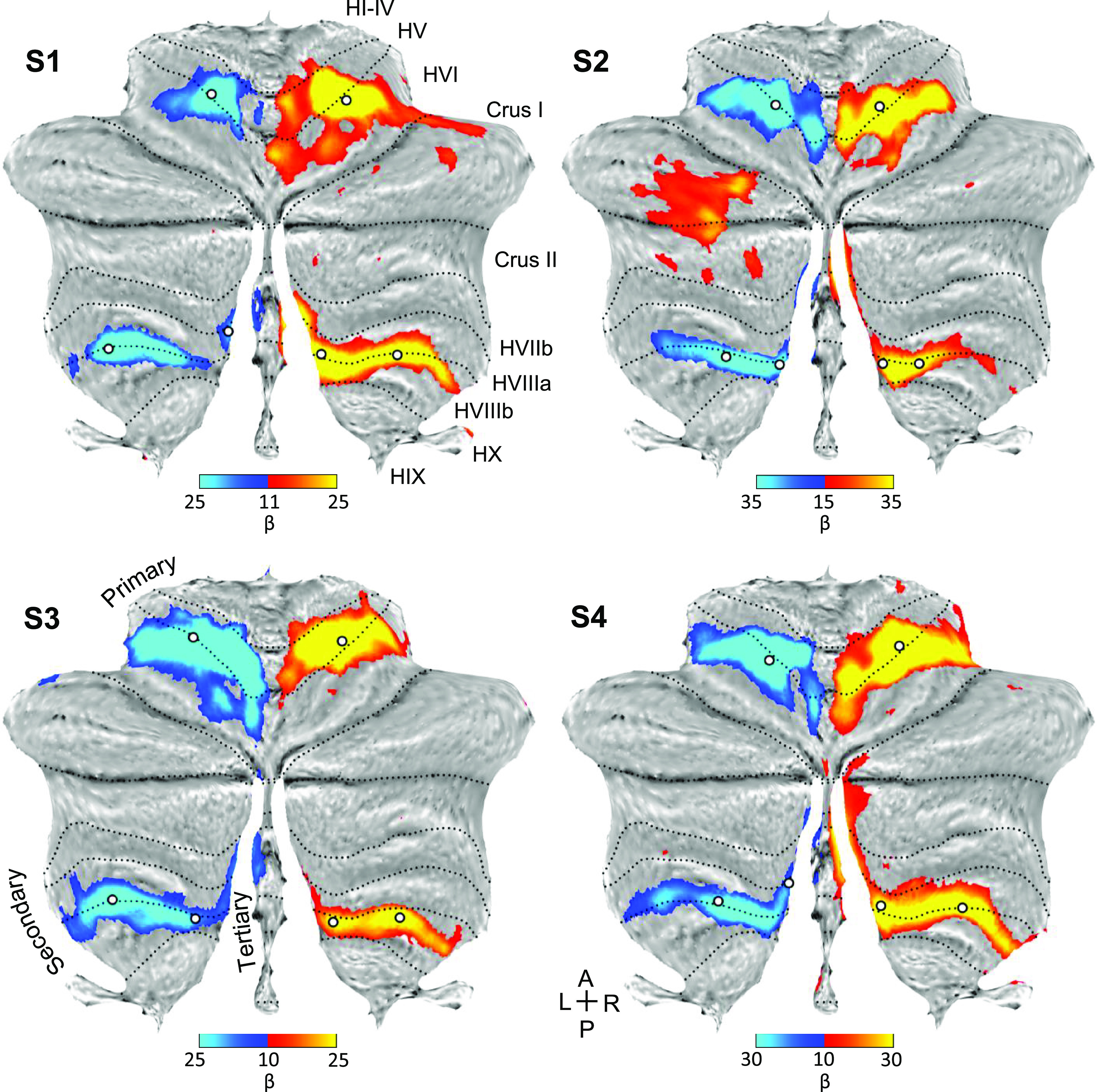
Direct contrast of left and right hand movements isolates representations of the body part. Contrast maps of right (red) versus left (blue) hand movements are projected onto flatmaps. Each panel displays a separate participant (*S1*–*S4*). In each participant, the hand representation is robust in both the anterior and posterior lobes. On the vermis, medial to the second representation, there is a bilateral hand representation in all participants. This extension of the hand representation may be a component of the tertiary representation, but it is ambiguous because there is no discontinuity. The white circles display the positions of seed regions that were used for analyses displayed in Fig. 5 (see text). Dotted lines indicate approximate lobule boundaries with lobules labeled for *S1*. A, anterior; L, left; P, posterior; R, right. Color bars indicate β values.

Contrasting right and left hand movements yielded robust responses in the cerebellum for all participants (Fig. 3). The primary and secondary representations were clear and distinct. In addition, all four individuals possessed bilateral hand representations on the edges of the vermis, medially and anteriorly to the second representation, potentially a component of the hypothesized third map. Specifically, in *S1* and *S2* there were two distinct representations for the second and the candidate third map. In *S3* and *S4*, the two representations appeared as one continuous cluster. Consequently, it is still ambiguous as to whether the medial representation is a distinct third representation or simply a continuation of the second representation. It seems that even if there is a third map, the hand representation is not positioned to provide clear evidence for dissociation. Therefore, we repeated the same analyses focused on the foot representation.

### The Foot Representation in the Cerebellum Differentiates the Third Somatomotor Map

**Figure 4.**
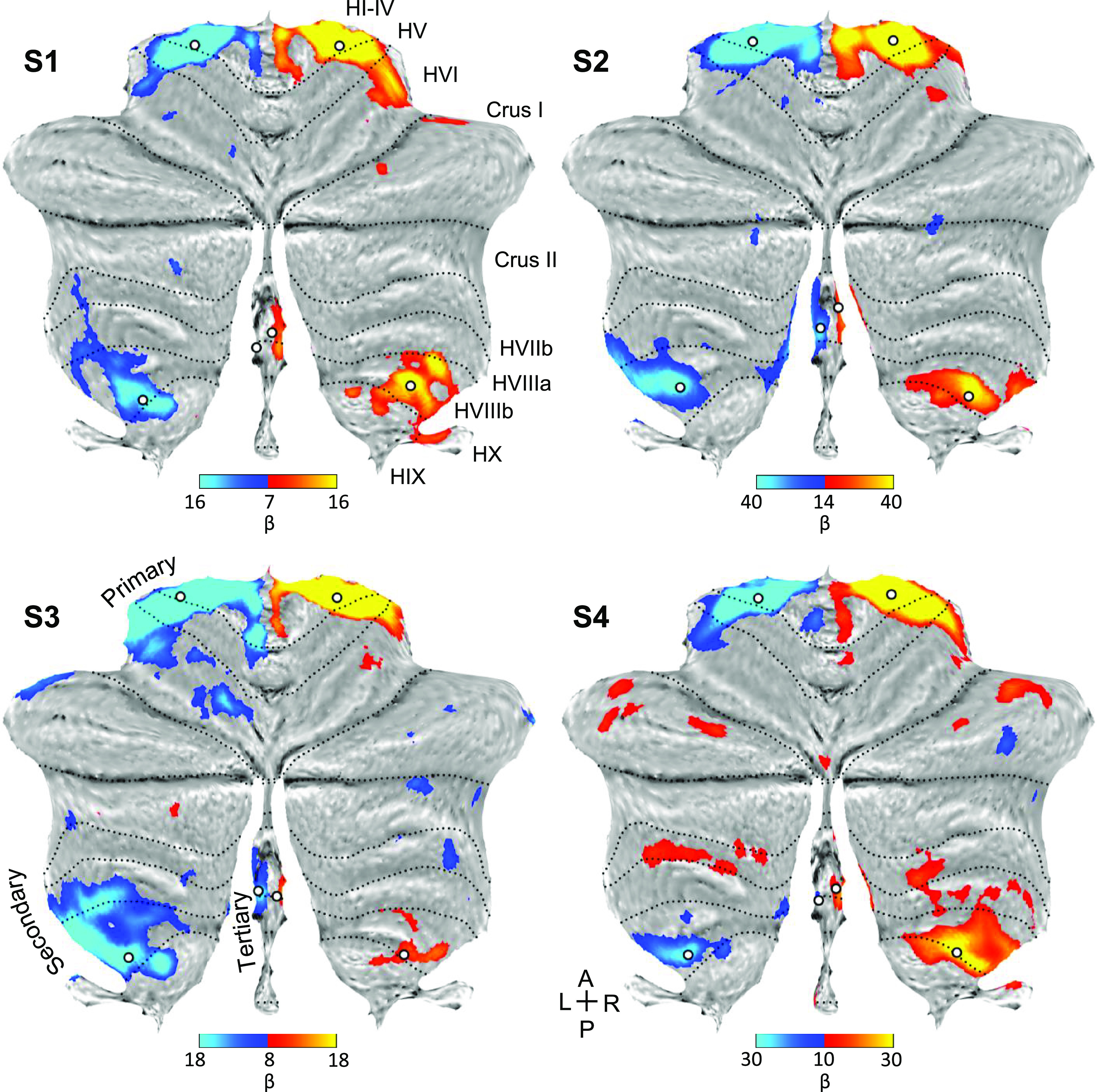
Direct contrast of left and right foot movements isolates three spatially discontinuous representations of the body part. Contrast maps of right (red) versus left (blue) foot movements are projected onto flatmaps. Each panel displays a separate participant (*S1*–*S4*). In each participant, the foot representation is detected in the anterior lobe and also the posterior lobe near to where the beginning of the secondary map is localized (laterally in lobule HVIIIb). Within the vermis, there is a distinct representation of the foot in each participant that is particularly clear in *S2* and *S3*. This spatially discontinuous representation is evidence for a third somatomotor map. The white circles display the positions of seed regions that were used for analyses displayed in Fig. 6 (see text). Dotted lines indicate approximate lobule boundaries with lobules labeled for *S1*. A, anterior; L, left; P, posterior; R, right. Color bars indicate β values.

Contrasting right and left foot movements yielded robust responses that included a representation in the vermis medial to the second representation in multiple participants (Fig. 4). Unlike the hand representation (Fig. 3), where the posterior lobe representation was continuous, the two representations associated with movement of the foot were spatially distinct (Fig. 4). One large representation was centered in lobule HVIIIb where the established second map’s upright topography begins. The smaller (newly) detected foot representations were medial and discontinuous with the known second somatomotor map. The presence of an independent foot representation in the vermis of the posterior lobe directly supports a third somatomotor map.

### The Third Cerebellar Somatomotor Map Shows Anatomically Specific Coupling to Cerebral Motor Cortex

Another way to refute or support the presence of independent cerebellar maps is to examine the specificity of cerebellar functional connectivity to the cerebral cortex. The positioning of the second and third somatomotor maps is such that *1*) the spatially separate cerebellar foot representations should both couple to the same midline cerebral zone (see Fig. 1), and *2*) the hand representations, which sit between the two foot representations in the cerebellum, should couple to a distinct cerebral zone near the hand knob of the precentral gyrus (see Fig. 1).

To explore these hypotheses, we first examined the specificity of the hand representations by applying seed-based functional connectivity analysis to the cerebellum and examining the correlation pattern in the cerebral cortex. Three sets of right and left seed regions were defined in the primary, secondary, and candidate tertiary representations (white dots, Fig. 3). Given that the obtained posterior lobe hand representation does not possess a discontinuity to separate the second from the third maps, the seed regions were estimated to be at or near the transitions to the foot representations, but still within the hypothesized hand representation.

**Figure 5.**
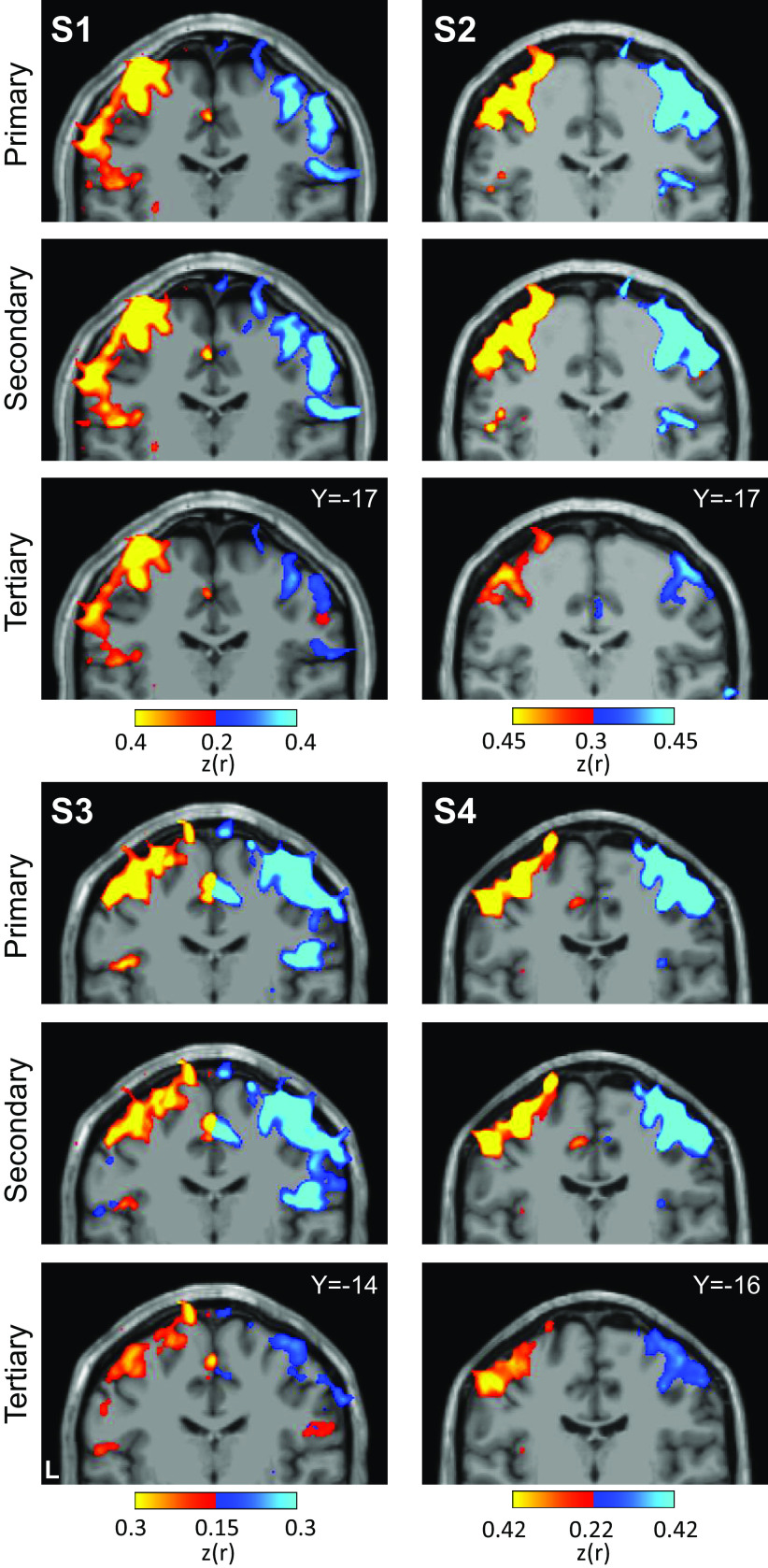
Seed-based functional connectivity of cerebellar hand representations reveals the contralateral hand region in M1. Coronal sections display functional connectivity patterns for hand seed regions in the right (red) and left (blue) cerebellum. In each column of three panels, an individual participant’s data are shown for separate sets of right versus left hand region contrasts that are independently seeded in the three cerebellar representations. The locations of the seed regions are illustrated by white circles in Fig. 3. In each individual, functional connectivity resulting from the estimated primary, secondary, and candidate tertiary cerebellar representations reveals M1’s contralateral hand region, demonstrating specificity. Note how, despite differences in correlation strength, the pattern revealed by the tertiary representation’s seed regions recapitulates the same pattern as the seed regions placed in the primary and secondary representations. Coordinates indicate the section level in the space of the MNI152 atlas. The color bars indicate correlation strength [*z*(*r*)]. L indicates left.

In all individuals, functional connectivity for all three seed region pairs revealed spatially specific cerebral regions at or near the hand region of M1 (Fig. 5). Right cerebellar seed regions correlated with the left cerebral hand region and left seed regions with the right. The results for the tertiary seed regions were found to be the weakest but possessed the same, spatially convergent pattern.

**Figure 6.**
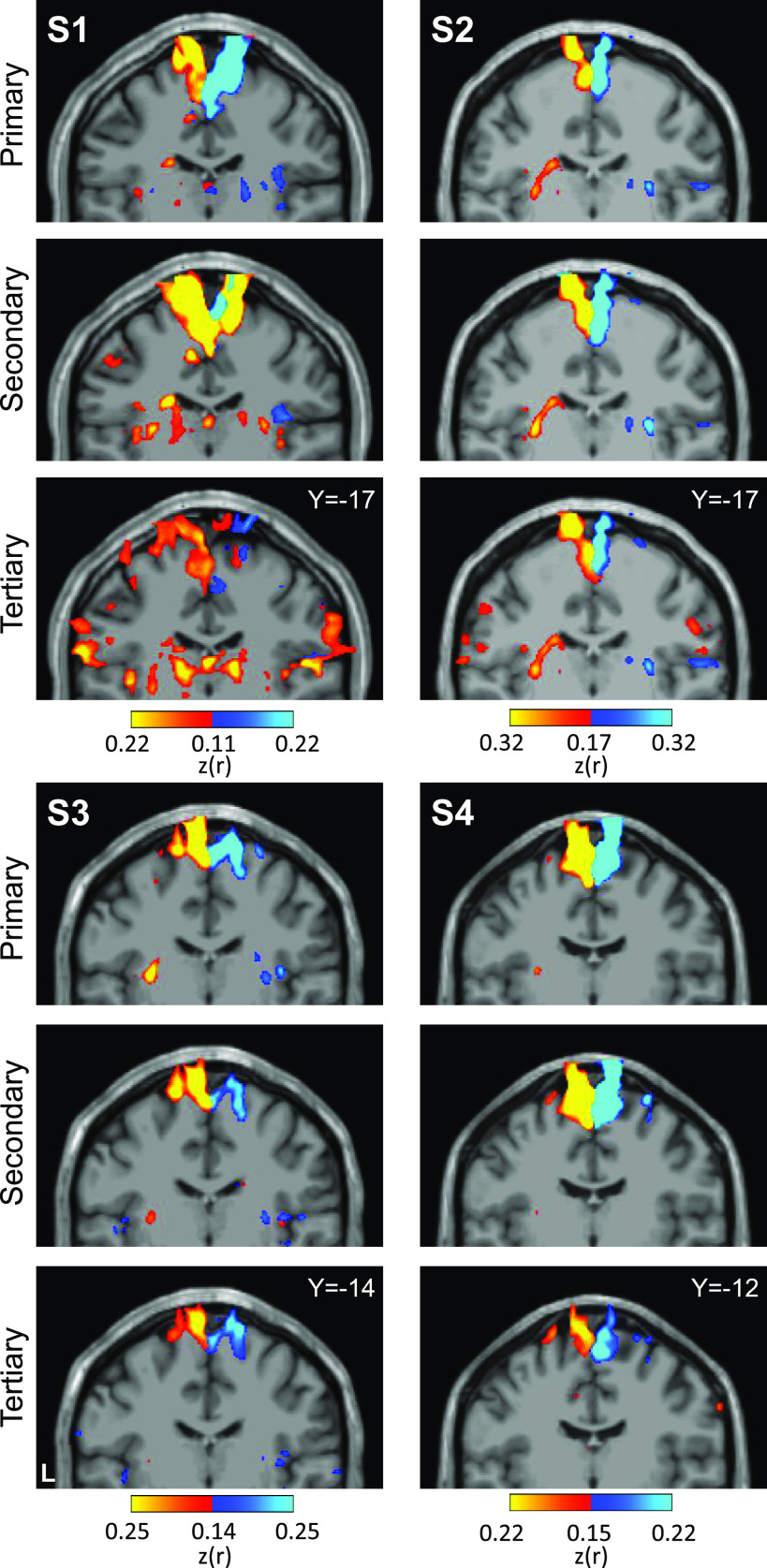
Seed-based functional connectivity of cerebellar foot representations reveals the contralateral foot region in M1, including for the spatially discontinuous tertiary representation. Coronal sections display functional connectivity patterns for foot seed regions in the right (red) and left (blue) cerebellum. In each column of three panels, an individual participant’s data are shown for separate sets of right versus left foot region contrasts that are independently seeded in the three cerebellar representations. The locations of the seed regions are illustrated by white circles in Fig. 4. In each individual, functional connectivity resulting from the estimated primary, secondary, and candidate tertiary cerebellar representations reveals M1’s contralateral foot region, demonstrating specificity. Note the pattern revealed by the tertiary representation’s seed regions recapitulates the same pattern as the seed regions placed in the primary and secondary representations despite being spatially discontinuous. Coordinates indicate the section level in the space of the MNI152 atlas. The color bars indicate correlation strength [*z*(*r*)]. L indicates left.

Applying a parallel analysis to the foot representations revealed a distinct pattern (Fig. 6). Unlike the hand representations, the seed region pairs for the second representation and candidate third representation of the foot are spatially discontinuous. Functional connectivity for all three seed region pairs revealed cerebral regions at or near the foot region of M1 that were distinct from the hand region. Of note, the hypothesized third foot cerebellar representation when seeded was, in isolation, able to recapitulate the selective cerebral motor pattern. This is important evidence because the zone of the cerebellum between the second and third maps is coupled to the cerebral hand region. The second and third foot representations are thus functionally selective and robustly dissociated from the cerebellar zone between them.

**Figure 7.**
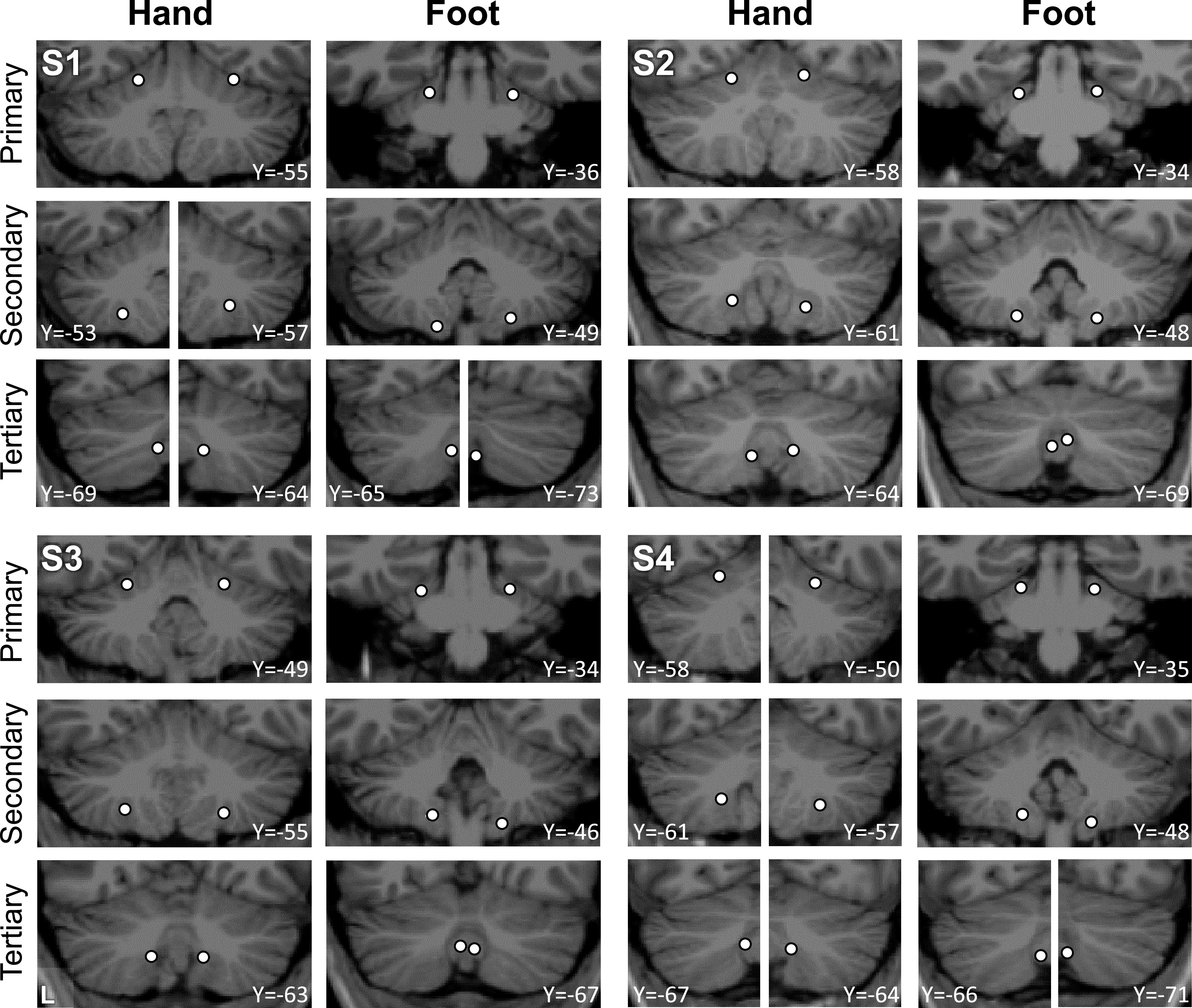
Visualization of seed region locations in the volume. Seed region locations of the right and left primary, secondary, and tertiary representations (white circles) are plotted on coronal sections of the T1 structural image for each participant. Sections presented are of the seed region’s center or within 1 mm of the center. For each individual, hand and foot seed region locations are presented. Note how the tertiary foot location is medial to the hand representation that separates it from the secondary foot location in each participant. Coordinates indicate the section level in the space of the MNI152 atlas. L indicates left.

To appreciate the discontinuity of the second and third somatomotor maps, the locations of the seed regions used in the aforementioned analyses were plotted on each individual’s structural volume image of their cerebellum. Seed regions for the second and third foot representations are on either side of the seed regions for the hand representation (Fig. 7). This observation alleviates concerns that the discontinuity could be an artifactual byproduct of the surface projection.

### Prospective Replication of the Third Cerebellar Somatomotor Map

The findings from the Discovery sample (*S1*–*S4*) reveal strong evidence for a third somatomotor map in the cerebellum. Given the importance of this observation and also that we have previously failed to find clear evidence for a third map,^1^ we sought to replicate all of the observations in an independent set of new participants. Critically, none of the Replication sample data (*S5* to *S8*) were processed until all of the analyses on the initial Discovery sample (*S1* to *S4*) were completed and plotted.

**Figure 8.**
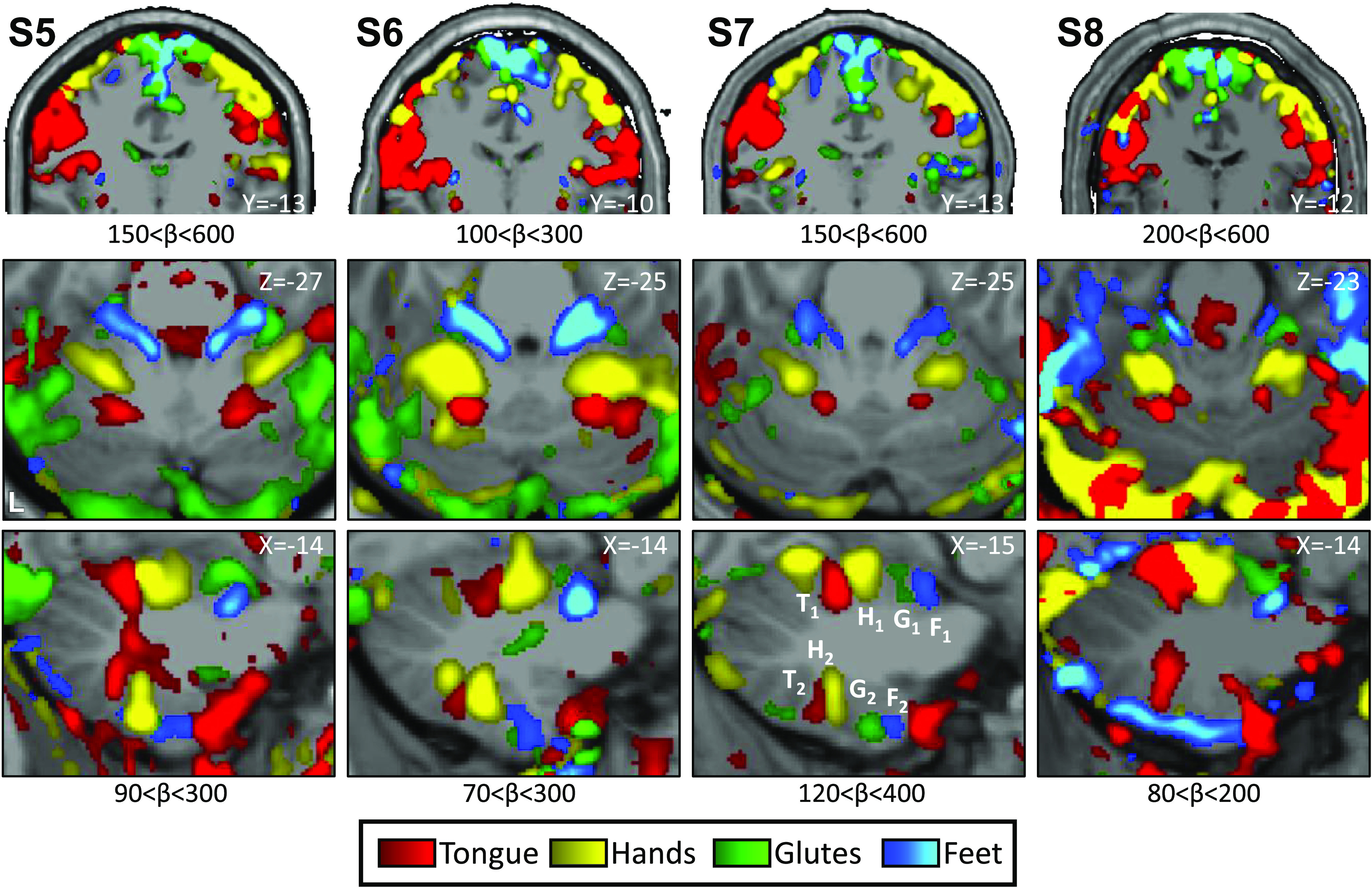
Replication of somatomotor topography in the cerebral cortex and cerebellum. Winner-take-all maps of active movements are displayed for four body parts along the anterior-posterior body axis: tongue (red), right and left hands (yellow), glutes (green), and right and left feet (blue). Each column displays a separate participant from the Replication sample (*S5*–*S8*). Analysis and plotting of these participants occurred after the results of the Discovery cohort were finalized. β values are thresholded to best capture topography, separately for the cerebral cortex and cerebellum. In each participant, a clear body topography is evident by the order tongue-hand-glutes-foot in the primary motor cortex, M1 (lateral to medial, *top row*), and cerebellar anterior lobe (*middle and bottom rows*). The primary cerebellar representation (T1-H1-G1-F1) is inverted to the secondary representation as labeled in *S7* (T2-H2-G2-F2). *S6* and *S7* show a full topography also in the cerebellar posterior lobe (bottom row) while a partial topography is observed in *S5* and only the tongue representation in *S8*. Coordinates indicate the section level in the space of the MNI152 atlas. The color bars represent β values. L indicates left.

**Figure 9.**
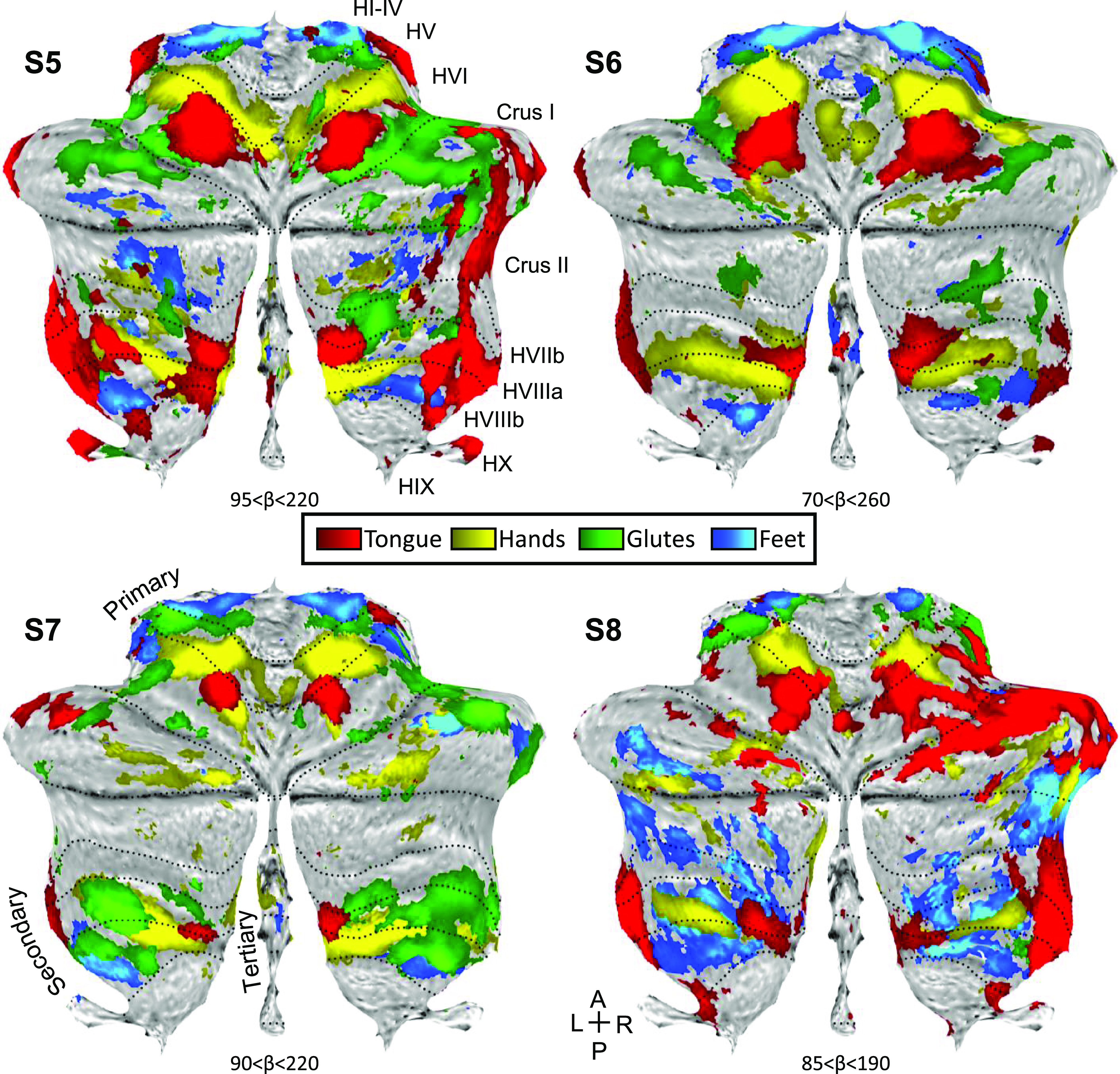
Replication of somatomotor topography projected onto a flatmap. Winner-take-all maps of active movements are displayed for four body parts along the anterior-posterior body axis projected onto flatmaps using the SUIT toolbox: tongue (red), right and left hands (yellow), glutes (green), and right and left feet (blue). Each panel displays a separate participant from the Replication sample (*S5*–*S8*). In participants *S5*–*S7*, the primary and secondary somatomotor maps are apparent in the anterior and posterior lobes of each hemisphere corresponding to the anterior-posterior body axis, with *S6* and *S7* showing the clearest examples. The experiment largely failed in *S8* (the primary map is apparent but the secondary map is ambiguous). Also note the medial representations within the vermis of *S5*–*S7*. These may be hints for the hypothesized tertiary body representation in the cerebellum. Dotted lines indicate approximate lobule boundaries with lobules labeled for *S5*. A, anterior; L, left; P, posterior, R, right.

In the Replication participants, we again found evidence for full body topography in M1 (Fig. 8, *top*) and in the cerebellar anterior lobe of all four individuals with the expected order of the tongue-hand-glutes-foot (Fig. 8, *middle* and *bottom rows*; Fig. 9). The second cerebellar map in the posterior lobe was also found with its full topography in *S6* and *S7*. *S5* and *S8* were missing clear representation of the glutes and were found to be generally noisier. In participants *S5*, *S6*, and *S7*, responses consistent with a third map were also evident with foot representations present within the vermis (Fig. 9).

**Figure 10.**
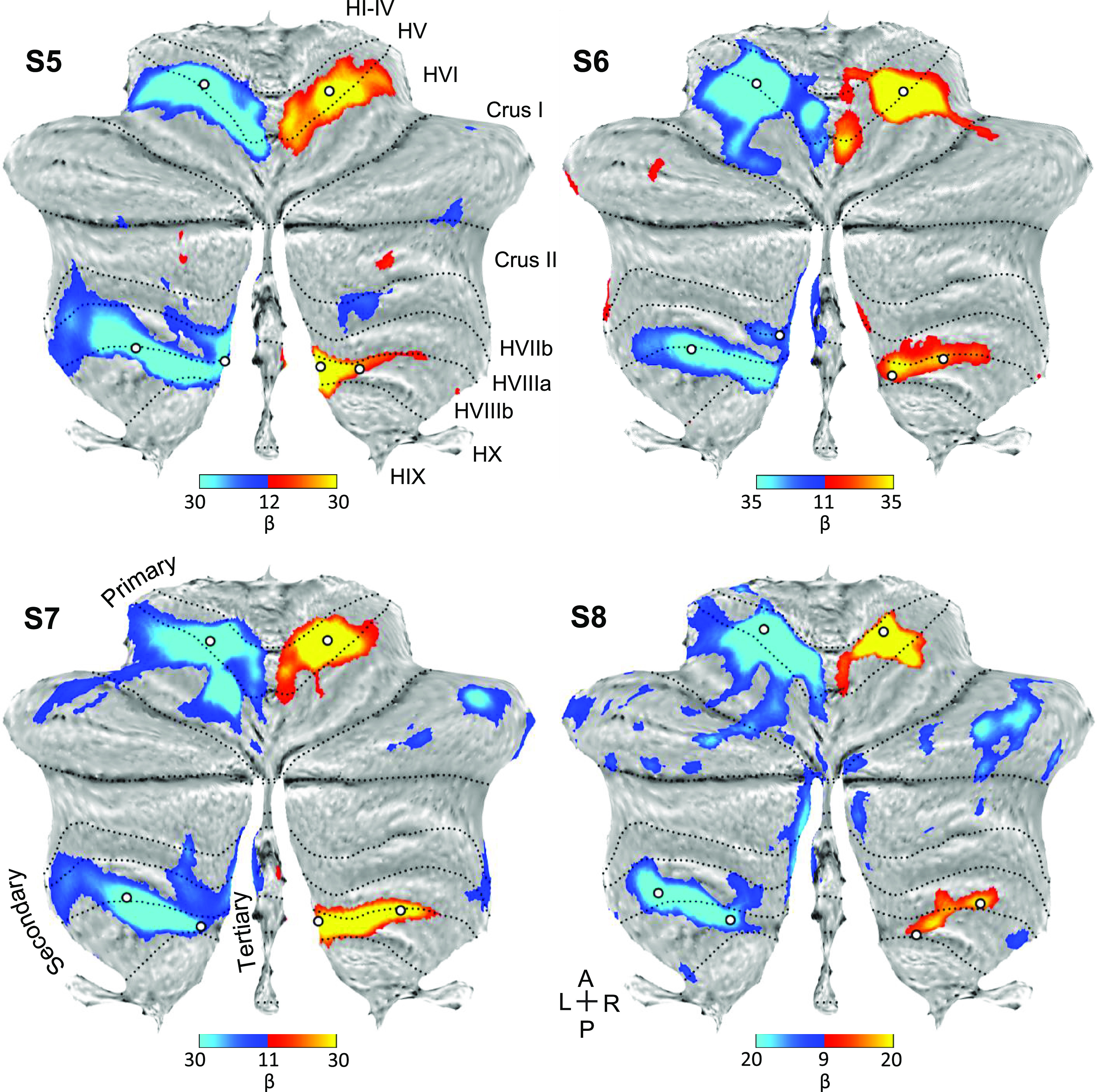
Replication of the hand representations. Contrast maps of right (red) versus left (blue) hand movements are projected onto flatmaps. Each panel displays a separate participant from the Replication sample (*S5*–*S8*). In each participant, the hand representation is robust in the anterior and the posterior lobes. The white circles display the positions of seed regions that were used for analyses displayed in Fig. 12 (see text). Dotted lines indicate approximate lobule boundaries with lobules labeled for *S5*. A, anterior; L, left; P, posterior; R, right. Color bars indicate β values.

**Figure 11.**
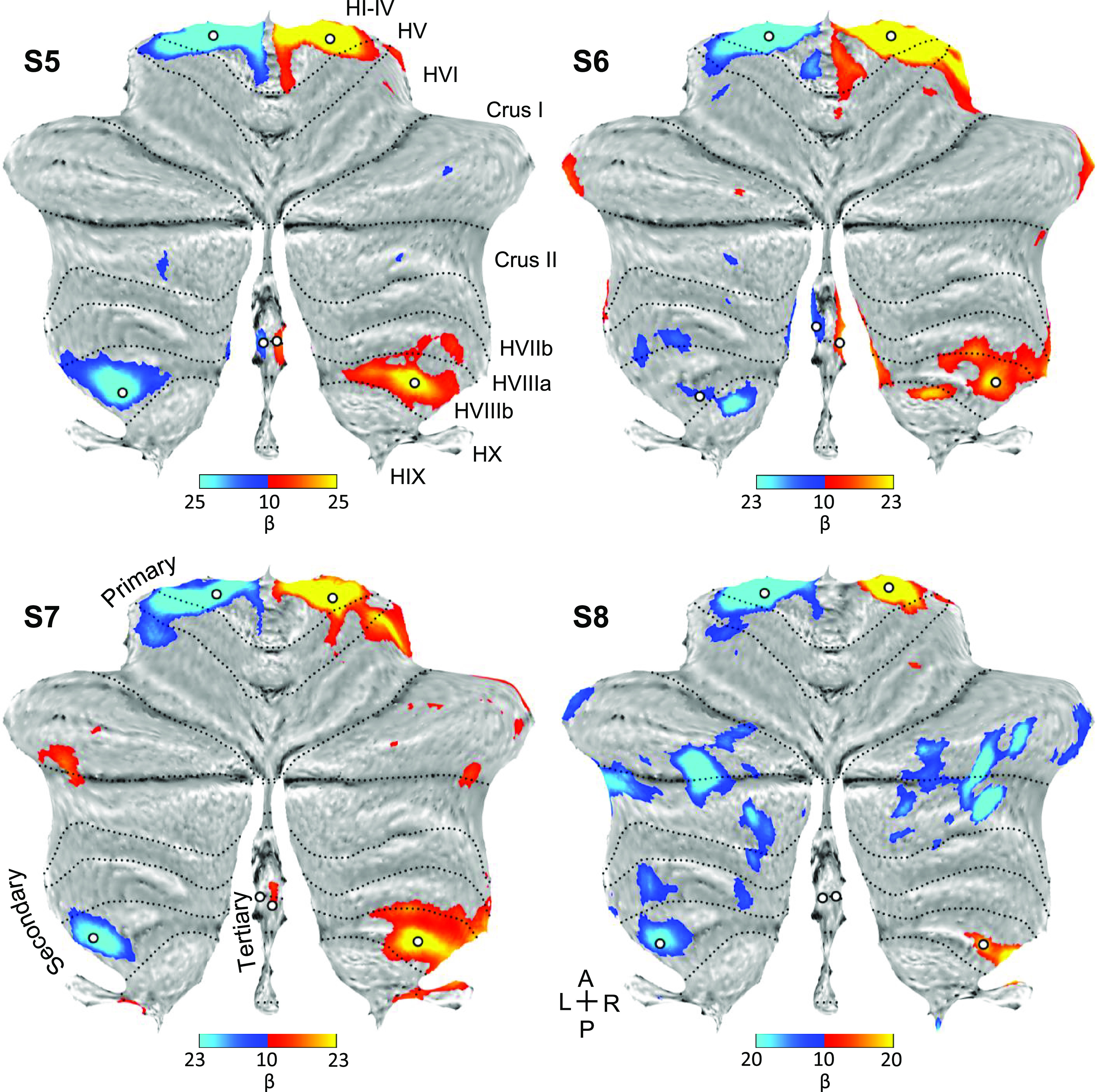
Replication of three spatially discontinuous foot representations. Contrast maps of right (red) versus left (blue) foot movements are projected onto flatmaps. Each panel displays a separate participant from the Replication sample (*S5*–*S8*). In three individuals, three separate foot representations can be identified—right and left primary, secondary, and a tertiary representation in the vermis (only right in *S7*). In *S8*, a tertiary representation was not detected. The spatially discontinuous representation within the vermis replicates evidence for a third somatomotor map. The white circles display the positions of seed regions that were used for analyses displayed in Fig. 13 (see text). Dotted lines indicate approximate lobule boundaries with lobules labeled for *S5*. A, anterior; L, left; P, posterior; R, right. Color bars indicate β values.

Contrasting right and left hand movements again yielded robust responses in the cerebellum (Fig. 10). The maps of the hand representations were present in all participants. Repeating the same analysis for foot movements yielded evidence for the third somatomotor map with discontinuous representation of the foot within the vermis (Fig. 11). *S5* and *S6* were particularly clean examples in the Replication sample, similar to the *S2* and *S3* in the prior Discovery sample. *S8* showed a great deal of nonspecific noise throughout the cerebellum and also lacked a clear foot representation within the vermis. Thus, across the two studies, there were multiple robust demonstrations of the third foot representation, but also the occasional ambiguous result and one clear failure (*S8*), consistent with prior studies of within-individual precision mapping that do not always achieve robust results in every individual (e.g., see Refs. 38 and 39).

**Figure 12.**
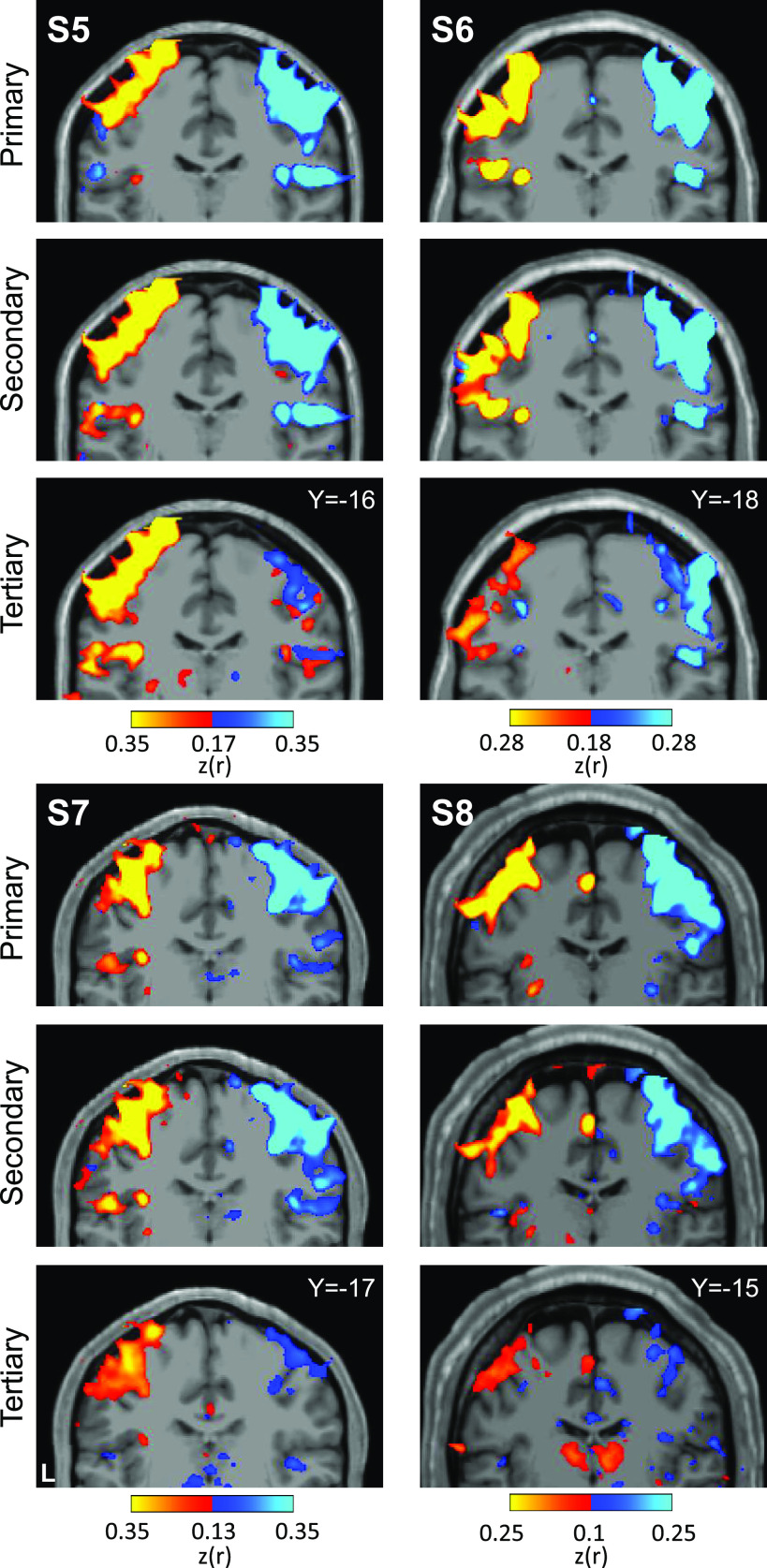
Replication of seed-based functional connectivity from cerebellar hand representations. Coronal sections display functional connectivity patterns for hand seed regions in the right (red) and left (blue) cerebellum. In each column of three panels, an individual participant’s data from the Replication sample are shown for separate sets of right versus left hand region contrasts that are independently seeded in the three cerebellar representations. The locations of the seed regions are illustrated by white circles in Fig. 10. Functional connectivity resulting from the estimated primary, secondary, and candidate tertiary cerebellar representations reveal M1’s contralateral hand region in all participants, demonstrating specificity. Note how, despite differences in correlation strength, the pattern revealed by the tertiary representation’s seed regions again recapitulate largely the same pattern as the seed regions placed in the primary and secondary representations. Coordinates indicate the section level in the space of the MNI152 atlas. The color bars indicate correlation strength [*z*(*r*)]. L indicates left.

**Figure 13.**
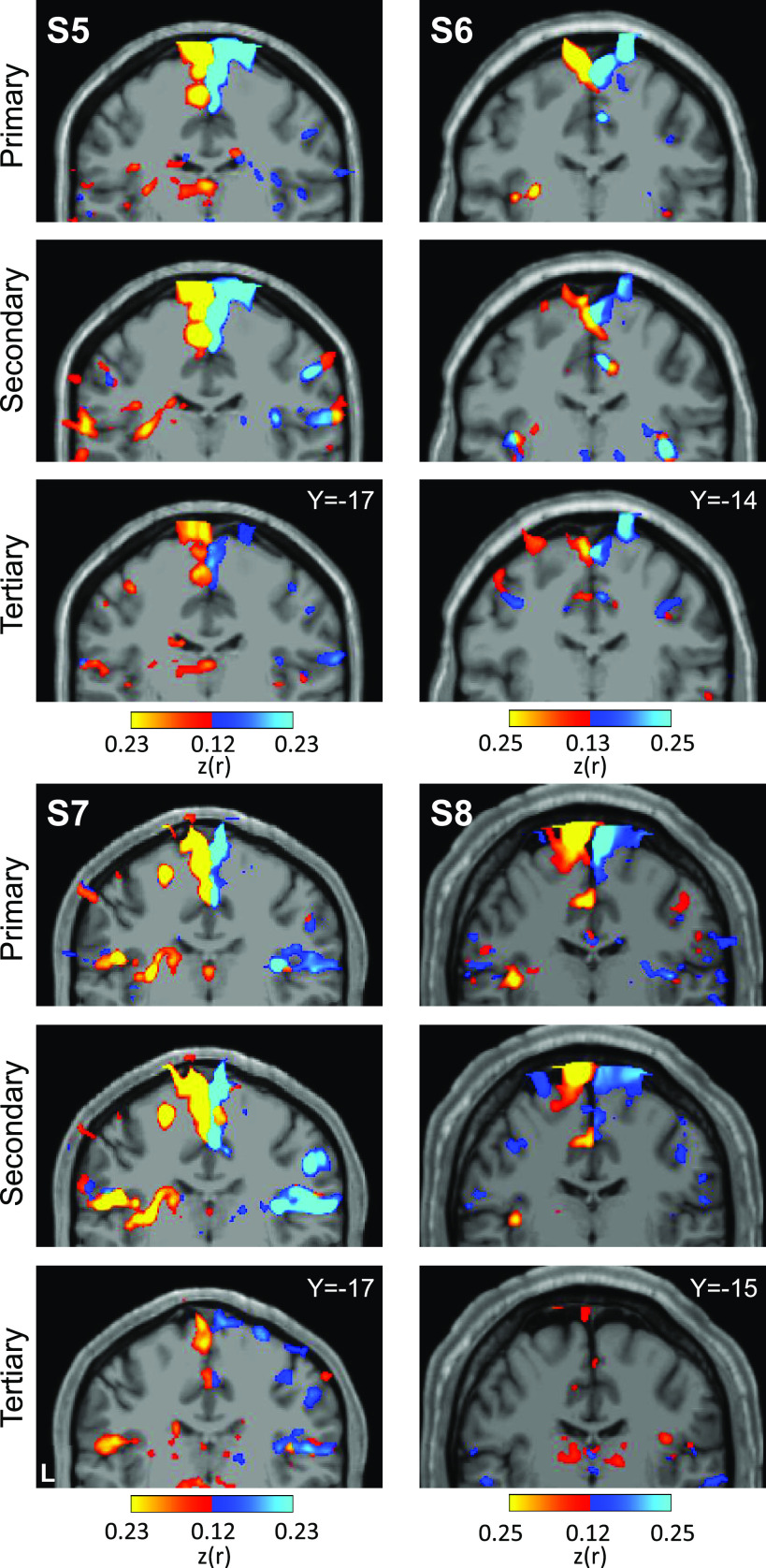
Replication of seed-based functional connectivity from cerebellar foot representations. Coronal sections display functional connectivity patterns for foot seed regions in the right (red) and left (blue) cerebellum. In each column of three panels, an individual participant’s data from the Replication sample are shown for separate sets of right vs. left foot region contrasts that are independently seeded in the three cerebellar representations. The locations of the seed regions are illustrated by white circles in Fig. 11. In three individuals (*S5*–*S7*), functional connectivity of seed regions in the primary, secondary, and candidate tertiary cerebellar representations reveal M1’s contralateral foot region (with *S5* and *S6* clearer than *S7*). The tertiary pattern is absent in *S8*. Coordinates indicate the section level in the space of the MNI152 atlas. The color bars indicate correlation strength [*z*(*r*)]. L indicates left.

**Figure 14.**
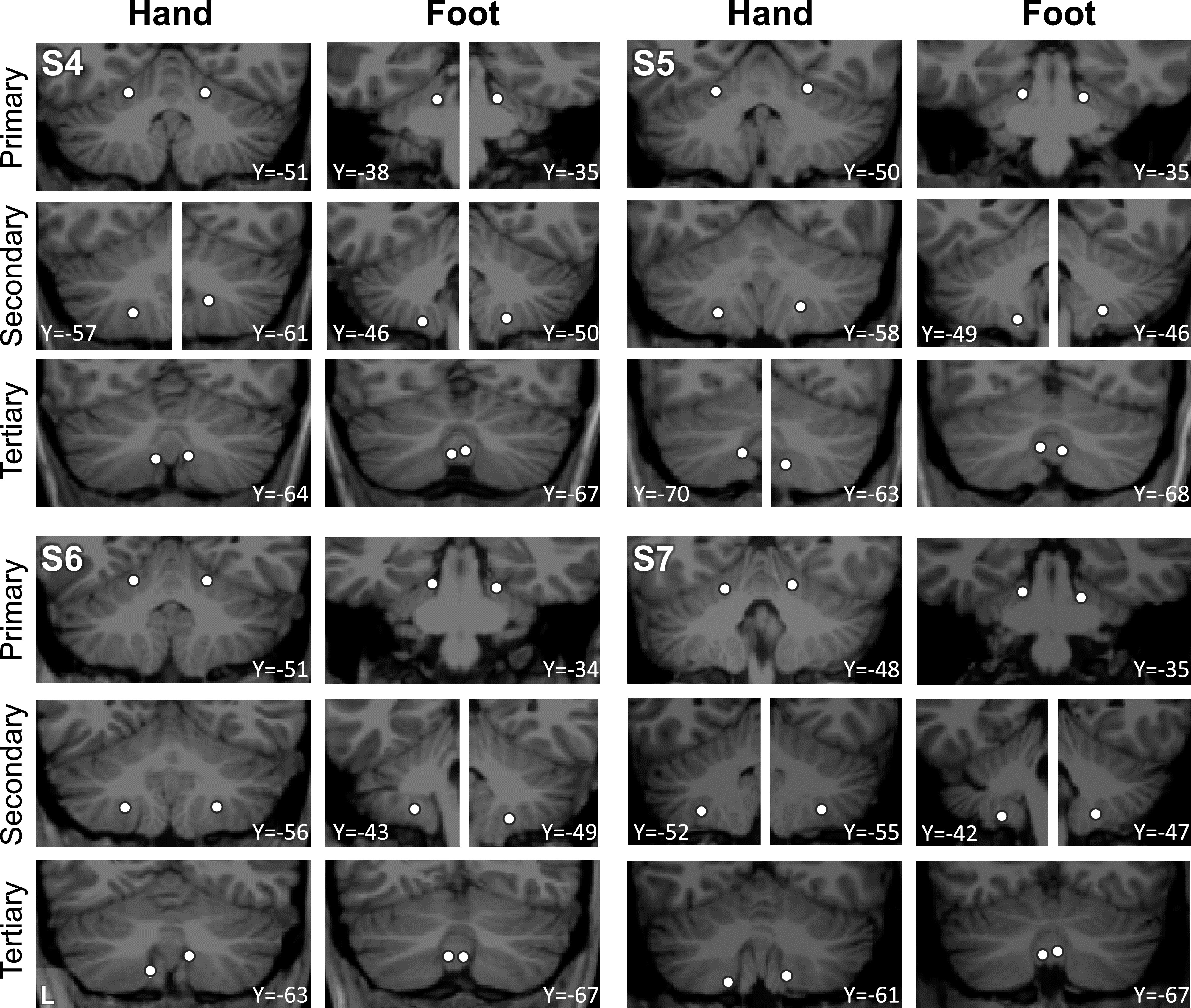
Visualization of seed region locations in the volume for *S5* to *S8*. Seed region locations of the right and left primary, secondary, and tertiary representations (white circles) are plotted on coronal sections of the T1 structural image for each participant in the Replication sample. Sections presented are of the seed region’s center or within 1 mm of the center. For each individual, hand and foot seed region locations are presented. Note how the tertiary foot location is medial to the hand representation in each participant. Coordinates indicate the section level in the space of the MNI152 atlas. L indicates left.

Placing seed regions within each of the three cerebellar maps again revealed the expected anatomically specific correlation patterns with cerebral motor zones (Figs. 12 and 13). High specificity was found in three individuals for all three representations. *S8*’s connectivity pattern showed M1’s bilateral foot region from the primary and secondary seed regions but failed to demonstrate evidence for a tertiary representation. The locations of the seed regions are displayed in the volume (Fig. 14), again highlighting the discontinuity of the second and third somatomotor representations.

### The Three Somatomotor Maps Are Evident When Examined at the Group Level

**Figure 15.**
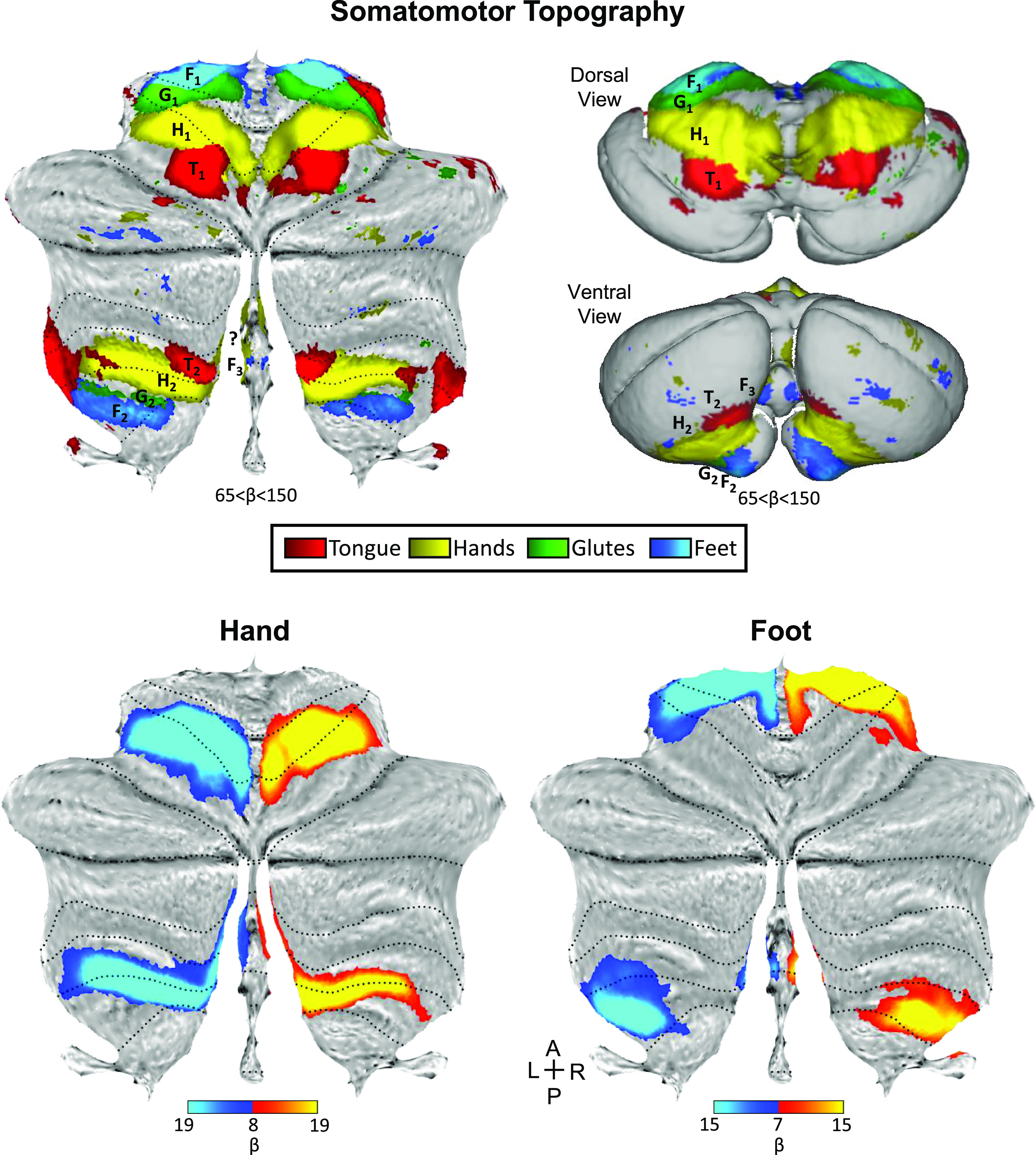
The three somatomotor representations are detected in the group-averaged maps projected onto the cerebellar surface. *Top*: group winner-take-all maps of active movements, generated after averaging contrasts across all eight individuals are displayed for four body parts projected onto a flatmap (*left*) and inflated pial surface (*right*): tongue (red), right and left hands (yellow), glutes (green), and right and left feet (blue). The top inflated surface shows a dorsal/anterior view of the cerebellum and the bottom surface shows the ventral/posterior view. The primary inverted body map is apparent in the anterior lobe and the secondary map in the posterior lobe. Note that the angle of the inflated ventral view hides the glutes representation in the secondary map (more visible on the flatmap and stronger on the left). Note candidate tertiary foot representations within the vermis, specifically in the ventral/posterior view of the inflated surface. *Bottom left*: a contrast map of right (red) versus left (blue) hand movements, averaged across participants, is projected on a cerebellar flatmap. The map is the mean contrast map of all eight participants including 185 separate runs collected during active movements. Dotted lines indicate lobular boundaries, as labeled in Fig. 2. Note the robust separation of the primary and secondary representation of the hand, with the secondary representation extending to the vermis. *Bottom right:* a parallel contrast map of right (red) and left (blue) foot movements is displayed. Note that there are three separate representations of the foot, with a tertiary representation falling within the vermis. The tertiary representation is spatially discontinuous with the secondary representation. The color bars represent the mean β values. A: anterior; L, left; P, posterior; R, right.

Although our studies were designed with the goal of preserving idiosyncratic anatomical details within the individual, the results suggested that there may be sufficient positional stability of the three somatomotor maps to examine the data at the group level. Figure 15 (*top*) shows the full somatomotor topography across all eight individuals (combining all data from the Discovery and Replication samples). A winner-take-all map was generated after averaging each movement contrast across individuals and projected onto flat map (*left*) and inflated cerebellar pial surface (*right*) visualizations. The primary and secondary somatomotor maps clearly present inverted and upright body topography correspondingly, suggesting that averaging data across participants can be effective. The posterior vermis shows hints of the tertiary map, clearer on the inflated surface, with the foot representation separated from the two others.

**Figure 16.**
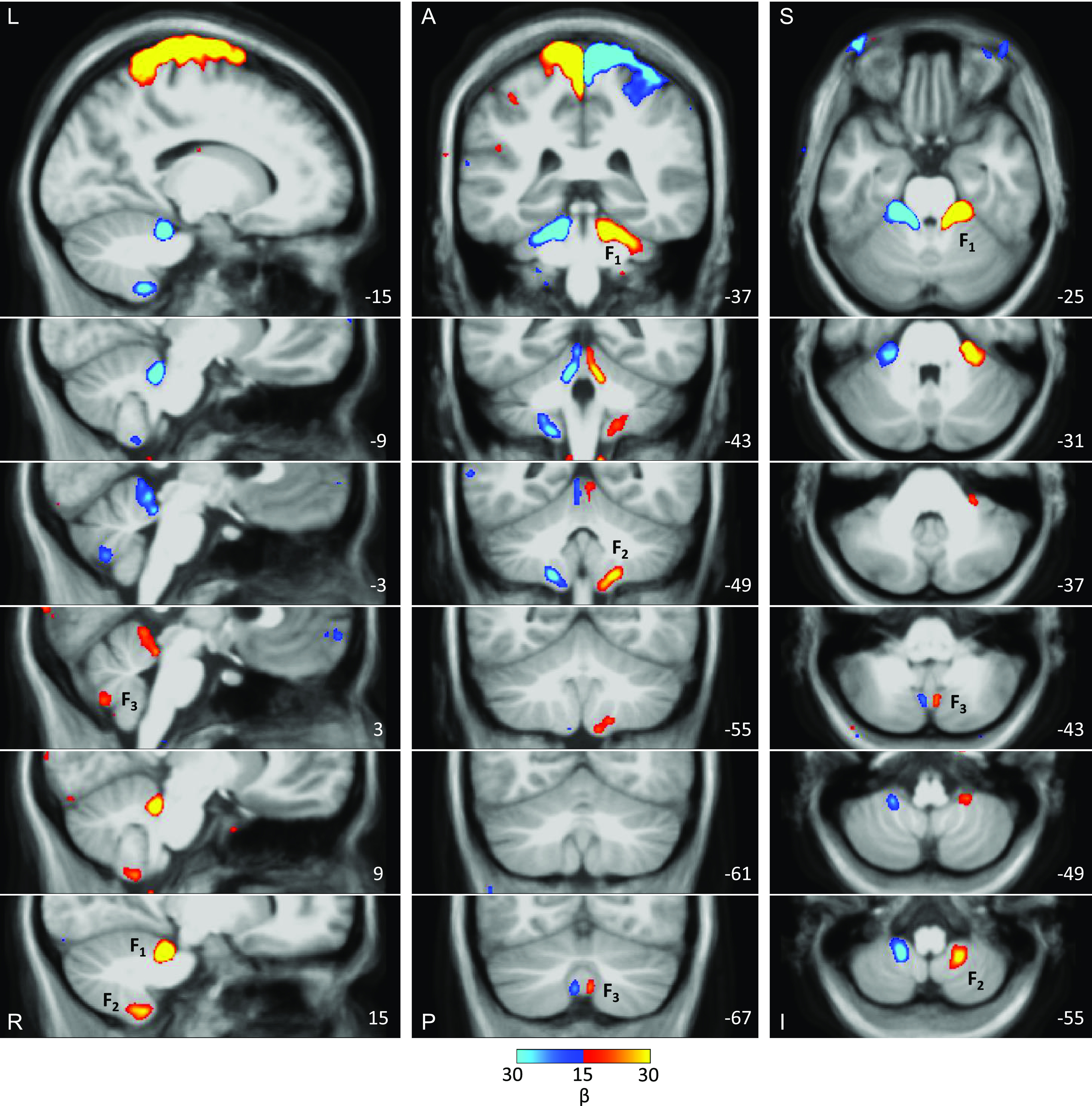
The three somatomotor representations of the foot are anatomically distinct in the group-averaged map, overlaid on the volume of the cerebellum. The three columns display sections in sagittal (*left*), coronal (*middle*), and transverse (*right*) orientation. In each orientation, the third foot representation is detected and anatomically separated from the second foot representation. The right primary, secondary, and tertiary representations are marked by F_1_, F_2_, F_3_. Note that the third representation is smaller in size as compared with the other two representations. The anatomical backdrop is the average volume from the eight participants contributing to the functional data. In the *middle* and *right columns*, left is displayed on the left. Coordinates at the *bottom right* of each panel indicate the section level in the space of the MNI152 atlas. The color bar represents the mean β values. A, anterior; I, inferior; L, left; P, posterior; R, right; S, superior.

Figure 15 (*bottom*) also shows the left versus right contrasts of the hand and foot movements averaged across all individuals. Critically, the small discontinuous responses linked to the right and left foot movements are evident within the vermis as would be expected from the analyses of the individuals. Once the location is known, it is now possible to see evidence in the group data. Figure 16 illustrates the location of the third representation of the foot in the group-averaged volume (marked by F_3_) along with the primary (F_1_) and secondary (F_2_) representations. The positioning and small size of the response also suggest why the third map representation is challenging to detect (and notice) as separate. We will return to this point in the discussion where the existing literature was reexamined for prior overlooked evidence for the third map.

**Figure 17.**
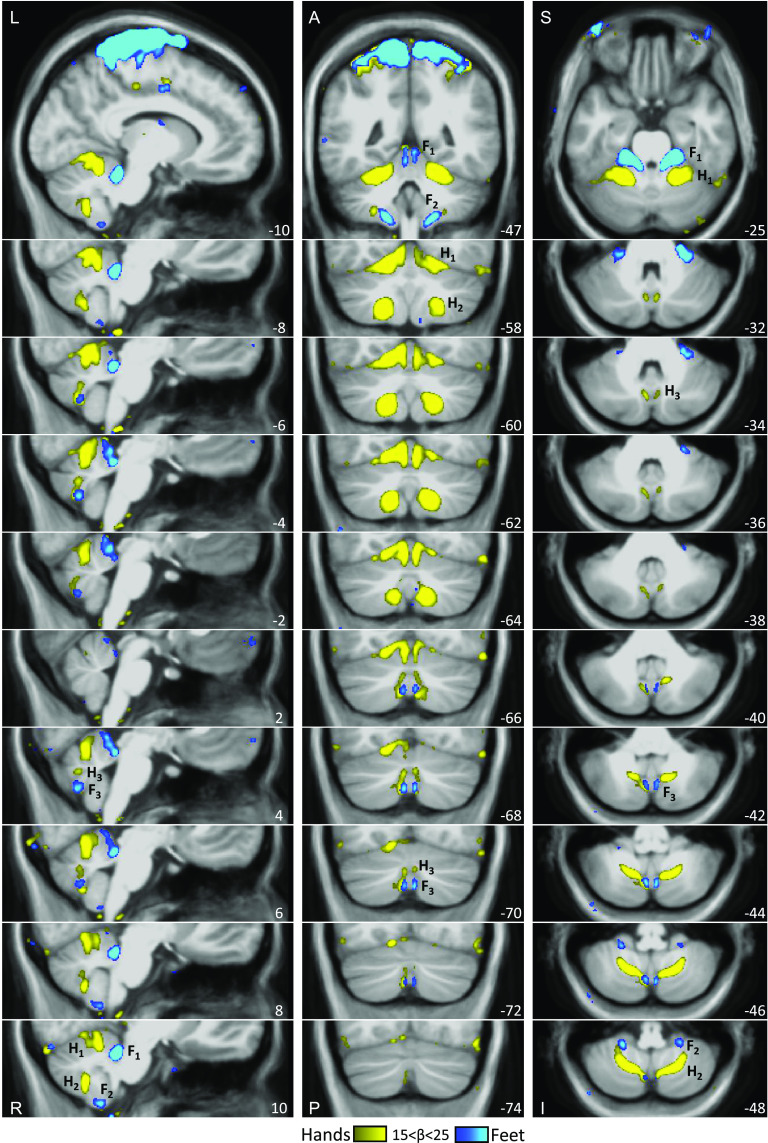
Relative location of hand and foot representations in the candidate tertiary map. The three columns display sections in sagittal (*left*), coronal (*middle*), and transverse (*right*) orientation. Right versus left contrasts show hand and foot primary (H_1_, F_1_), secondary (H_2_, F_2_), and tertiary (H_3_, F_3_) representations. Note that the tertiary hand representation is superior and lateral to the foot. It is unclear what the definitive orientation of the tertiary body map is since the secondary and tertiary hand representations appear here as continuous. Nonetheless, these data are consistent with an upright map. The anatomical backdrop is the average volume from the eight participants contributing to the functional data. In the *middle* and *right columns*, left is displayed on the left. Coordinates at the *bottom right* of each panel indicate the section level in the space of the MNI152 atlas. The color bar represents the mean β values. A, anterior; I, inferior; L, left; P, posterior; R, right; S, superior.

For another perspective on the relative location of the hand and the foot representations of the candidate tertiary map, Fig. 17 shows a group averaged contrast across multiple 2-mm-spaced slices. The tertiary hand representation appears superior and lateral to the foot. Still, because the secondary and hypothesized tertiary hand representations are spatially continuous, we do not have sufficient information to definitively determine the third body map orientation. Nonetheless, the orientation of the hand and foot representations in Fig. 17 is suggestive of an upright map.

## DISCUSSION

Our data provide evidence for a third somatomotor representation within and near to the vermis of the human cerebellum. Unlike the two well-established somatomotor maps that can be distinguished as separate by exploring the hand, foot, or other body parts bilaterally, the linchpin for detecting the separation between the second map and candidate third map was examining the multiple representations of the foot. When left and right foot movements were contrasted, a response was detected in the cerebellar vermis that was spatially discontinuous from the known second map. Moreover, the region of the posterior cerebellum between the two foot representations was associated selectively with the hand, providing evidence that the two foot representations are parts of spatially- and functionally distinct maps. All findings were detected in multiple independent participants and replicated in a second, prospectively analyzed cohort. We discuss these observations in the context of what they reveal about the broad topography of the cerebellum as well as implications for the specific organization of the vermis.

### Evidence for the Elusive Third Cerebellar Somatomotor Map

Although two somatomotor maps within the cerebellum have long been established (1–3; for review see Ref. 40), it has been difficult to find clear evidence for a third map.^1^ Guell et al. (15, 19) hypothesized that a third somatomotor map may not exist, suggesting an organizational distinction between how motor zones of the cerebellum are represented in contrast to nonmotor zones. By their “double motor/triple nonmotor representation hypothesis,” the most posterior zones of the cerebellum are occupied only by regions associated with higher-order cognitive and affective functions. The present results suggest that three distinct somatomotor maps may be present in the cerebellum, with the third falling at or near the third representation of nonmotor networks.

There are multiple possible reasons why the third representation has been challenging to identify. First, it is small and buried in a difficult to access and hard to image region of the cerebellum. Second, studies using human neuroimaging have tended to focus on hand and finger movements and less on direct contrasts of foot movements. For example, the recent work by King et al. (24) that produced detailed, comprehensive maps of cerebellar functional zones in both groups and individuals included only finger movement paradigms and did not include foot movements. We suspect if detailed analyses of foot movements had been more common in the field, consensus about the representation may have already emerged.

A third challenge deserving deeper discussion is that task-free functional connectivity estimates of cerebellar somatomotor representations have been surprisingly equivocal. As one example from our own recent work where we explored two individuals each scanned across 31 sessions (20), the somatomotor organization was the weakest aspect of the data. Even after revisiting that data based on the present findings, we cannot clearly establish the second map by contrasting left and right motor regions linked to the cerebral foot representations, despite there being little debate about the existence of the second map. The third map, being smaller than the second, is not detected. As another example, in our studies at high field (e.g., see Ref. 27) we were not able to detect robust functional connectivity between cerebral motor zones and the cerebellum in the face of clear functional connectivity from higher-order networks including the default network. In the recent work of Marek et al. (28) that examined within-individual estimates of cerebellar organization from extensive functional connectivity data, the second somatomotor map is variably absent. This can be seen in individuals but also in their averaged flatmap representation (see Fig. S2 in Ref. 28). Although there is a clear inverted topography in the anterior lobe, posterior lobe cerebellar regions within the vicinity of the established second somatomotor map are assigned to higher-order cognitive networks including the default network. We suspect that functional connectivity between the cerebral cortex and the cerebellum, as examined in our multiple earlier studies and also in these other studies in the literature, is yielding an incomplete estimate of somatomotor organization. We do not fully understand the origins of this limitation but have been perplexed previously on multiple occasions.^1^

In this light it is interesting to revisit the results reported in Guell et al. (15) because their study is one of the few to examine cerebellar organization using both task-free functional connectivity and also task-evoked active motor movements. Using data from the Human Connectome Project (41), the study is well-powered, including data from 787 participants with the ability to directly contrast left and right active foot movements. What is notable is that the group-averaged task-evoked estimates in Guell et al. (see Fig. 4 in Ref. 15) are highly similar to our present group-averaged map (Fig. 15), with a spatially discontinuous representation of the foot in the posterior vermis. The estimate of the foot representation from the task-free data does not show the same pattern as the task-evoked estimate. Thus, when examined closely, the present results converge with earlier studies that have examined task-evoked somatomotor topography.

### Contextualizing the Third Somatomotor Map within the Broader Organization of the Cerebellum

The cerebellum possesses multiple roughly homotopic representations of the full cerebrum (13, 19–21). Two sets of somatomotor, cognitive and affective networks are found that begin in the anterior lobe and progress along the anterior-to-posterior axis. A primary representation spans from the anterior extent of the cerebellum to Crus I/II, and a secondary representation spans from Crus I/II to the posterior extent of the somatomotor representation.^2^ Yet, a parsimonious account of cerebellar organization as multiple repeating cerebral representations encounters an inconsistency.

Evidence for a third partial representation of cerebral networks has been found posterior to the second somatomotor representation (13, 15, 20, 24). The third representation includes cognitive and affective regions, but the expected somatomotor component that would complete the representation has been missing. The present finding of a third foot representation, localized to the posterior vermis (Figs. 15 and 16), is consistent with the possibility that the cerebellum possesses three separate representations of the full cerebrum, each including a topographic representation of somatomotor cortex.

The location of the third map within the vermis is particularly intriguing because this region has been understudied in humans despite being a major focus or work in animal models (e.g., see Refs. 42–47). Recently, van Es et al. (48) demonstrated that the human cerebellar vermis responds to visual stimuli. Detailed within-individual analysis confirmed this observation and further noted that the region is coupled to primary retinotopic visual cortex (20). The third foot somatomotor representation is spatially near to the visual representation within the vermis, suggesting that multiple sensory and motor maps may be proximal to one another in this small buried region of the cerebellum, and also near to representations of non somatomotor cerebral networks, including those important to affective function (e.g., see Refs. 49 and 50). This juxtaposition of sensory and motor maps between domains is not predicted by a simple model in which all aspects of cerebellar topography are understood within a continuous homotopic mapping of the cerebrum, as visual cortex is distant from somatosensory and motor cortices in the cerebrum. Further study of the organization and functional importance of the human vermis is thus of great interest.

### A Gap Remains

Our data provide direct evidence for three separate discontinuous representations of the foot. Based on the relative location of hand and foot representations within the vermis, we speculate that the tertiary body map is likely upright, similar to the secondary map, where the hand representation is anterior to the foot. Unlike the secondary representation, the tertiary hand representation appears lateral to the foot. However, despite mapping four body parts extensively within each individual participant, our results do not provide definitive evidence for the orientation of the third map. There are multiple possible reasons for this gap. First, the tertiary representation is likely smaller than the other two. It is possible that the spatial resolution used here is not sufficient for mapping the full topography, especially given the complex surface topology of the cerebellum (51). Second, the tongue and the glutes are more susceptible to head motion, and therefore the corresponding representations are noisier than the ones for the hands and feet, which also allow direct subtraction of left and right movements. Future explorations with intensive within-individual sampling at higher resolution will be informative. In addition, exploring a somatosensory paradigm where lateralized stimulation (right vs. left) can be administered repeatedly across multiple body parts along the anterior-posterior axis could allow for additional contrasts (similar to hands and feet here) and may provide a path toward better understanding of the tertiary map orientation.

In the current study, we used a task of simple motor movements to assess the cerebellar somatomotor maps. It was previously suggested that complex movements (e.g., sequences of individual digits flexion and extension) can result in a different pattern of cerebellar activity and engage regions that are not activated during simple movements (52). The present paradigm is not able to map responses related to complex movements or speculate about a specific functional role of the tertiary map. Future explorations might also benefit from including complex movements in the experimental paradigm.

### Conclusions

We provide reliable evidence that the human cerebellum possesses three spatially distinct representations of the foot. The location of the third foot representation is in the vermis, consistent with a third somatomotor map continuous with the tertiary representations of cognitive and affective networks. The third map’s small size and location may have contributed to its evasiveness in past explorations, and suggests examination at high spatial resolution will be necessary to fully understand the third map’s spatial orientation and its relation to functionally distinct zones of the vermis.

## GRANTS

This study was supported by National Institutes of Health (NIH) Grant MH124004 and NIH Shared Instrumentation Grant S10OD020039. This work was also supported by the National Science Foundation Graduate Research Fellowship Program under Grant No. DGE1745303 (to L.M.D.). 

## DISCLAIMERS

Any opinions, findings, and conclusions or recommendations expressed in this material are those of the authors and do not necessarily reflect the views of the National Science Foundation.

## DISCLOSURES

No conflicts of interest, financial or otherwise, are declared by the authors.

## AUTHOR CONTRIBUTIONS

N.S.-G. and R.L.B. conceived and designed research; P.A.A. and L.M.D. performed experiments; N.S.-G., P.A.A., and L.M.D. analyzed data; N.S.-G., P.A.A., L.M.D., and R.L.B. interpreted results of experiments; N.S.-G. and R.L.B. prepared figures; N.S.-G. and R.L.B. drafted manuscript; N.S.-G., P.A.A., L.M.D., and R.L.B. edited and revised manuscript; N.S.-G., P.A.A., L.M.D., and R.L.B. approved final version of manuscript.

## Footnotes

1 Within our own work, we have previously looked for a third map using functional connectivity fMRI both at high-field, high-resolution 7 T (27) and within intensively studied individuals at 3 T (20). These efforts yielded equivocal results. Considering also Buckner et al. (13), the present effort is our fourth attempt to find evidence for a third somatomotor map.

2 We heuristically term the somatomotor/body maps as primary, secondary, and tertiary. As discussed by Guell et al. (15), this terminology is a heuristic for convenience and should not be taken to imply a known relation between the representations such as exists in primary and secondary visual cortical areas where direct thalamic input goes to the earlier (primary) area in a hierarchy of anatomically connected areas. The ordering of the somatomotor maps in the cerebellum reflects the order in which the representations were discovered, not a functionally meaningful hierarchy.
